# Modeling of Solid–Liquid Equilibria in Deep Eutectic Solvents: A Parameter Study

**DOI:** 10.3390/molecules24122334

**Published:** 2019-06-25

**Authors:** Ahmad Alhadid, Liudmila Mokrushina, Mirjana Minceva

**Affiliations:** 1 TUM School of Life and Food Sciences Weihenstephan, Technical University of Munich, Biothermodynamics, Maximus-von-Imhof-Forum 2, 85354 Freising, Germany; ahmad.alhadid@tum.de; 2 Friedrich-Alexander-Universität Erlangen-Nürnberg (FAU), Separation Science & Technology, Egerlandstr. 3, 91058 Erlangen, Germany; liudmila.mokrushina@fau.de

**Keywords:** deep eutectic solvents, solid–liquid equilibria, modeling phase equilibria, melting properties, activity coefficient models

## Abstract

Deep eutectic solvents (DESs) are potential alternatives to many conventional solvents in process applications. Knowledge and understanding of solid–liquid equilibria (SLE) are essential to characterize, design, and select a DES for a specific application. The present study highlights the main aspects that should be taken into account to yield better modeling, prediction, and understanding of SLE in DESs. The work is a comprehensive study of the parameters required for thermodynamic modeling of SLE—i.e., the melting properties of pure DES constituents and their activity coefficients in the liquid phase. The study is carried out for a hypothetical binary mixture as well as for selected real DESs. It was found that the deepest eutectic temperature is possible for components with low melting enthalpies and strong negative deviations from ideality in the liquid phase. In fact, changing the melting enthalpy value of a component means a change in the difference between solid and liquid reference state chemical potentials which results in different values of activity coefficients, leading to different interpretations and even misinterpretations of interactions in the liquid phase. Therefore, along with reliable modeling of liquid phase non-ideality in DESs, accurate estimation of the melting properties of their pure constituents is of clear significance in understanding their SLE behavior and for designing new DES systems.

## 1. Introduction

Deep eutectic solvents (DESs) are a class of eutectic mixtures of two or more compounds that have a eutectic point far below the melting temperatures of the individual components [1]. The large depression in the melting temperature of the mixture is commonly attributed to strong hydrogen bonding interactions between DES constituents. The recent trend toward green solvents has increased the potential of DESs in many applications [2,3,4,5]—e.g., as solvents for active pharmaceutical ingredients [6,7,8], fuel production [9,10], solid–liquid and liquid–liquid extraction [11,12,13,14,15,16], and solid–support free liquid–liquid chromatography [17,18,19,20].

Similar to ionic liquids (ILs), DESs are considered designer solvents because their properties can be tailored by choosing different combinations of its constituents [2]. However, the ratio of constituents—i.e., the eutectic composition—is not as easy to determine as it is for ILs, in which the ratio of cations and anions is determined by mixture electroneutrality. Many molecular simulation studies can be found in the literature; these aimed to understand DES systems at the molecular level and determine their eutectic composition [21,22,23,24,25,26,27,28,29,30,31,32,33,34,35]. It should be noted, however, that these studies were mainly performed using DESs of a fixed composition corresponding to a supposed hydrogen bond complex. Therefore, the studied compositions are not representative of the entire range of possible compositions. Even with fixed ratios between DES constituents, no specific microstructure was found, but rather a network of hydrogen bonding [35]. It has been postulated that the hydrogen bonding network structure could be correlated to eutectic temperature [34]. Molecular simulation studies are a helpful tool in modeling and understanding interactions between DES constituents at a molecular level, but they are so far unable to provide sufficient macroscopic information required for process applications. Although knowledge and understanding of solid–liquid equilibria (SLE) in DES systems are essential to designing and selecting the appropriate system for a particular application, only a small fraction of DES related literature contains information about their SLE [1,36,37,38,39,40,41,42,43,44,45,46,47].

Eutectic systems are mixtures of two or more components in which the components show complete or partial immiscibility in the solid state at the mixture melting temperature [48]. Although eutectic mixtures have been long studied, no agreement for strict criteria to distinguish between eutectic mixtures and “deep” eutectic mixtures can be found in literature [49]. In recent publications, the term DES or natural DES (NADES) is typically used for any mixture that forms a eutectic mixture with a low freezing temperature. Smith et al. [4] defined DESs as systems that have a large difference between the melting temperature of the mixture at eutectic composition and the weighted sum of the melting temperatures of the pure components. They assumed that the deep eutectic point is associated with the formation of a new complex via hydrogen bonding between DES constituents; therefore, the eutectic composition represents the stoichiometry of this complex. Based on this definition, the search for eutectic compositions has been commonly performed by trial and error using a specific ratio between constituents such as 1:1, 1:2, etc. Despite the fact that in some DES systems the eutectic composition represents a specific molar ratio between the constituents—such as 1 choline chloride ([Ch]Cl):2 urea or 1 [Ch]Cl:1 oxalic acid—the eutectic point of any phase diagram is determined by the intercept of the liquidus lines of the components, even if the solution behaves ideally. Therefore, complex formation might not be the only reason for deep eutectic formation and the eutectic composition may not accord with a specific molar ratio between the constituents.

Martins et al. [49] proposed a more comprehensive definition for DES systems. According to these authors, DES systems are mixtures of two or more components—i.e., not a new compound. At the same time, DES systems should be differentiated from other simple eutectic systems based on their strong negative deviations from ideality—i.e., the eutectic temperature is lower than that of the ideal eutectic. In addition, the term DES should not refer exclusively to eutectic composition but to any composition at which the mixture is liquid at operating temperature. Based on this definition, studying the non-ideality of the liquid phase is essential to determining whether or not a system is a DES. Following this, most recent literature studies on modeling SLE in DESs have focused mainly on modeling non-ideality in the liquid phase as a reason behind the formation of DESs [42,43,44,45,46,49,50]. From a thermodynamic perspective, SLE do not depend only on the intermolecular interactions in the liquid phase but also on a correct estimate of the reference state—i.e., the ratio of the fugacities of the pure components in the solid and subcooled liquid phases at system temperature. Although this was clearly and explicitly stated many years ago in Prausnitz et al. [51], it continues to be overlooked in the literature. Only consideration of the interplay of both pure component melting properties and solution non-ideality can result in reliable modeling of SLE in DESs, which will help in understanding their nature. Moreover, activity coefficients and melting properties are not independent since the value of the reference state chemical potential the activity coefficients refer to is directly related to the melting properties.

Although melting enthalpy and melting temperature can be measured using differential scanning calorimetry, it is still a difficult issue because of polymorphism, possible metastable solid phases, kinetic limitations due to high liquid viscosities close to the melting temperature, etc. For some substances, it is impossible to measure them owing to their thermal instability. These issues result in limited and inaccurate data, especially on the enthalpy of fusion. Despite the fact that the literature reports several theoretical methods for the estimation of melting properties, these methods usually suffer from many limitations and are hardly applicable to ionic compounds [52].

Recently, Kollau et al. [47] and Martins et al. [49] studied the effect of melting properties and non-ideality on SLE phase diagrams. Defining DES as mixtures with high negative deviation from ideality, they demonstrated that the SLE phase diagram should be known to distinguish DES from other eutectic mixtures and that the non-ideality in DESs can be quantitatively described by the regular solution theory or the PC-SAFT equation of state. However, the focus of these studies was to convince the community that only eutectic mixtures with high negative deviations from ideality should be considered as DES. A number of issues covered in the present study were not discussed or, if considered, then indirectly and not in detail.

The present work presents a systematic study of both melting properties and solution non-ideality, with the goal of understanding DES formation. Consideration is first made based on a hypothetical model eutectic system to find the link between eutectic point properties—eutectic temperature and composition—with both the melting properties and activity coefficients of the components. Then, simplified modeling of [Ch]Cl/ILs eutectic systems is carried out to evaluate the effect of small melting enthalpies on the interpretation of the modeling results. Further, DES systems composed of quaternary ammonium chlorides and fatty acids are modeled to evaluate the effect of overestimation of melting enthalpies of components and to support the initial findings. The only criteria for selecting the activity coefficient model used in this work—namely, Redlich–Kister polynomial—is its simplicity and flexibility in describing curves of different shape and complexity. It should be pointed out that this work does not propose or recommend this model to be used for SLE calculations in DES. The calculations done in this work do not aim at improved modeling of published SLE data; instead, the aim is to encourage discussion and further interpretation of these data.

The general objective of this work is to provide the deeper understanding essential to establishing the basic steps required in defining and designing DES systems based on their SLE behavior. Similar to molecular simulation approaches, thermodynamic modeling of SLE can be a valuable tool to assess the behavior of components in the liquid phase—i.e., intermolecular interactions. Designing DES systems based on their SLE estimated from activity coefficient models or equation of state might have an advantage over molecular simulation approaches owing to the direct estimation of eutectic temperature, eutectic composition, and the composition range at which the DES is a liquid at operating temperature.

## 2. Theory

**Figure 1:**
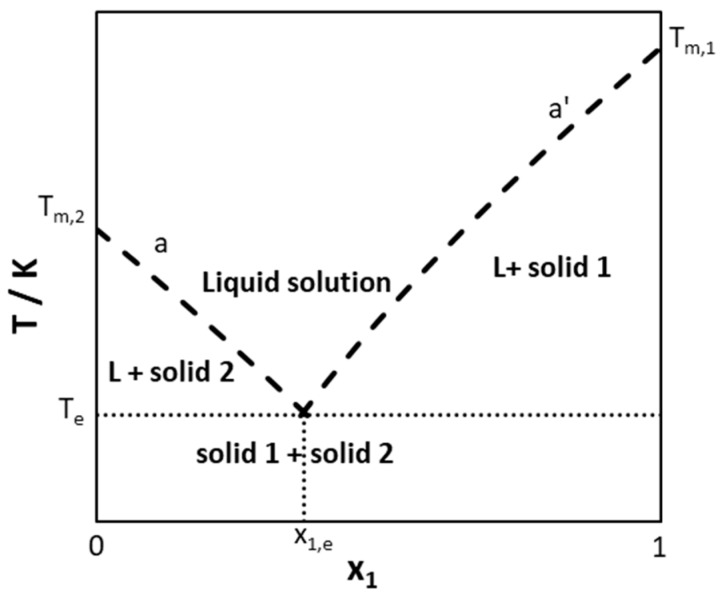
Schematic representation of a solid–liquid phase diagram of a simple eutectic system.

Most studied DESs have been considered as simple eutectic systems in which pure constituents crystallize in the form of pure components at the mixture liquidus temperature. Figure 1 shows a schematic of a solid–liquid phase diagram of a simple eutectic system. Curve (a’) and (a) are the liquidus lines of Component 1 and 2, respectively. They intercept at the eutectic point, which corresponds to the lowest melting temperature—the eutectic temperature (T_e_)—of a mixture of eutectic composition (x_e_).

The liquidus line of component i is calculated as [51]

(1) $$\ln x_{i}^{L}\gamma_{i}^{L}=\frac{\mu_{0i}^{S}-\mu_{0i}^{L}}{RT}=\ln\frac{f_{0i}^{S}}{f_{0i}^{L}}=-\frac{\Delta h_{m,i}}{RT}\left(1-\frac{T}{T_{m,i}}\right)-\frac{\Delta c_{P}}{R}\;\left(1-\frac{T_{m,i}}{T}+\ln\frac{T_{m,i}}{T}\right)$$

where $x_{i}^{L}$ and $\gamma_{i}^{L}$ are the mole fraction and activity coefficient of component i in the liquid solution, respectively; $\mu_{0i}^{L}$ and $\mu_{0i}^{S}$ are the reference chemical potentials of pure component i in the subcooled liquid and solid states at liquidus temperature, respectively; $f_{0i}^{L}$ and $f_{0i}^{S}$ are the reference fugacities of pure component i in the subcooled liquid and solid states at liquidus temperature, respectively; $\Delta h_{m,i}$ and $T_{m,i}$ are the melting enthalpy and the melting temperature of pure component i, respectively; T is the liquidus temperature; $\Delta c_{P,\;i}$ is the difference in heat capacity of pure component *i* in the solid and liquid states at constant pressure; R is the universal gas constant.

The $\Delta c_{P}$ term on the right-hand side of Equation (1) is typically of low value in comparison with the $\Delta h_{m,i}$ term. In addition, the $\Delta c_{P}$ values are usually unavailable from previous experiments and are difficult to estimate with reasonable accuracy because no liquid exists at temperatures below the melting temperature. As a result, the second term on the right-hand side of Equation (1) has been commonly neglected in literature and the following simplified equation is used to calculate the liquidus lines

(2) $$\ln x_{i}^{L}\gamma_{i}^{L}=-\frac{\Delta h_{m,i}}{RT}\left(1-\frac{T}{T_{m,i}}\right)$$

According to Equation (2), the position of the eutectic point, as well as the whole SLE phase diagram, depend on both the melting properties—melting enthalpy and melting temperature—of the pure components and their activity coefficients in solution. When the solution is assumed as ideal ($\gamma_{i}^{L}=1$), the SLE phase diagram can be modeled based on the melting properties of the pure components only. However, if the system deviates from ideal behavior, the activity coefficients of components in the liquid phase can be calculated using well-developed g^E^ models or equations of state, which are available in the literature.

## 3. Results and Discussion

### 3.1. Parameter Study on a Hypothetical Binary System

In the present section, a hypothetical binary DES system is considered. The high melting component is labeled as 1 and the low melting component is labeled as 2.

#### 3.1.1. Melting Properties of Pure Components

**Figure 2:**
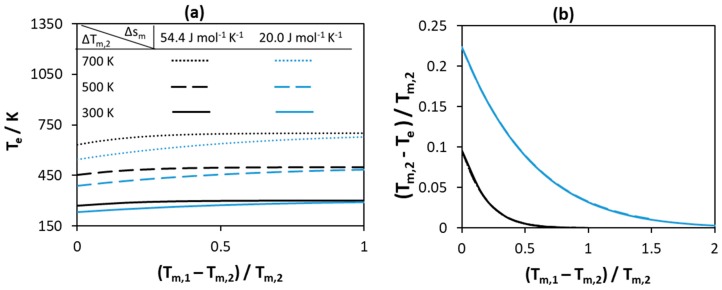
Effect of melting temperature of pure components with constant melting entropy on (**a**) the eutectic temperature (**b**) the normalized depression at eutectic temperature relative to that of the low melting Component 2.

**Figure 3:**
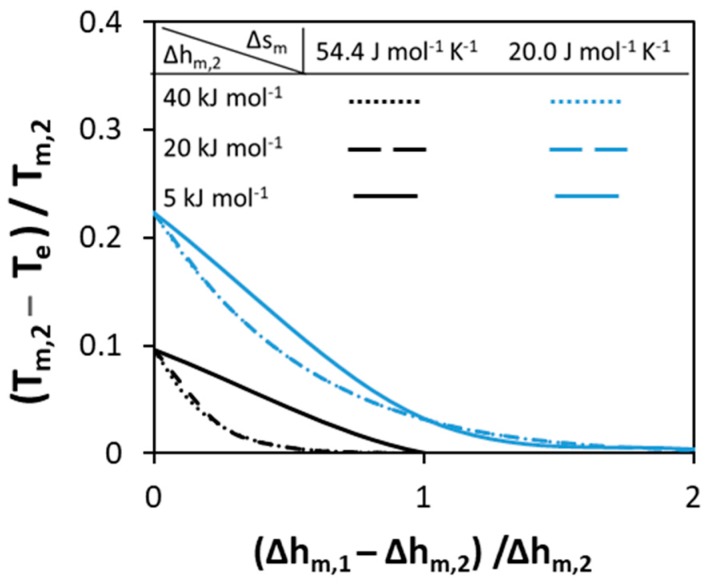
Effect of the melting enthalpies of pure components with constant melting entropy on the normalized depression at eutectic temperature relative to that of Component 2.

**Figure 4:**
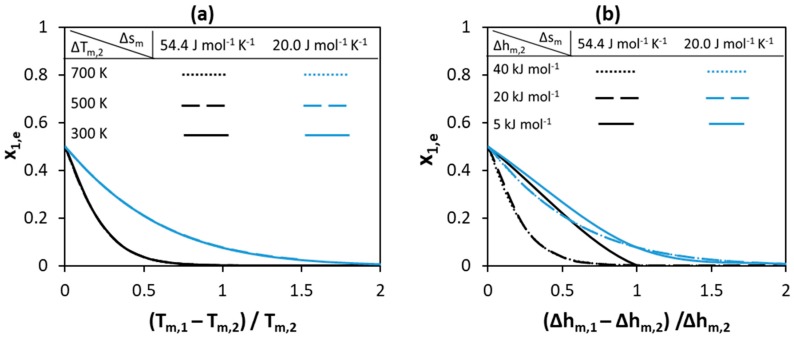
Effect of (**a**) melting temperature (**b**) melting enthalpy of pure components on the eutectic composition of Component 1.

The melting properties of the pure components—melting enthalpy and melting temperature—are prerequisites for SLE diagram calculations even if the solution is assumed ideal. Melting enthalpy and entropy depend on the molecular arrangement in the crystal lattice—i.e., molecular symmetry, conformational diversity, and intermolecular forces [52]. The melting temperature is the ratio between melting enthalpy and entropy of the component; therefore, a high melting temperature of a compound corresponds to a high melting enthalpy, a low melting entropy, or both [53]. Highly ordered solid structures usually have high melting temperatures as a result of a high melting enthalpy. However, disordered solid structures may also have high melting temperatures as a result of low melting entropy. In this case, the low melting entropy corresponds to association in the liquid phase—as a result of strong hydrogen bonding [54]—or due to a symmetrical and rigid molecular structure [53]. Therefore, it is very difficult to correlate the melting enthalpy values of compounds—or even to obtain an estimate based on melting temperature only—without information relating to (i) their crystal and molecular structures and (ii) interactions between like-molecules in the liquid phase. However, according to Bondi’s observation, melting entropy of components can be better related to the molecular structure [55], although it cannot be measured and is estimated from experimental melting enthalpy and temperature of compounds. Therefore, two cases are considered in this section to represent components of different crystal structures. In the first case, a high melting entropy is assumed of 54.4 ${J\;\mathrm{mol}}^{-1}K^{-1}$($\Delta s_{m}/R\approx 7$)—also known as the Walden rule (Equation (3))—to represent compounds with ordered crystals of rigid molecules [53]. In the second case, a low melting entropy of $20.0{\;J\;\mathrm{mol}}^{-1}K^{-1}$($\Delta s_{m}/R\approx 2.4$) is assumed to represent compounds with disordered crystals of rigid molecules [53]. In this section, the effect of melting properties on the eutectic point properties—eutectic temperature (T_e_) and composition (x_e_)—of a hypothetical ideal binary system is studied, with the results shown in Figure 2, Figure 3 and Figure 4. In all cases, SLE are modeled using Equation (2) assuming $\gamma_{i}^{L}=1$.

Figure 2 represents the effect of melting temperatures of pure components on the eutectic temperature of the system. As seen from Figure 2a, the absolute value of the eutectic temperatures changes as the melting temperature of the low and the high melting components changes. The higher the values of the melting temperatures of both components, the higher the eutectic point of the system. As the difference between the components increases, the absolute eutectic temperature increases and reaches a constant value that is close to the melting temperature of Component 2. The low value of melting entropy of the components at constant melting temperatures further decreases the eutectic temperature of the system—the blue curves in Figure 2a are always lower than the black curves. As seen in Figure 2b, if the normalized difference between the eutectic temperature and the melting temperature of the low melting component is selected as a measure for the depression at eutectic point, the absolute values of the melting temperatures of both components have no effect on the depression at eutectic point. This effect is a result of fixing the melting entropy of components—increasing the melting temperatures of components at constant melting entropy means an increase in the melting enthalpy of components. Therefore, the effect of melting temperatures of components is negligible if the melting entropy and similarly the melting enthalpy of components is high. Therefore, at constant value of melting entropy, the largest depression is observed if the melting temperatures of both components are equal.

In Figure 3, the effect of the melting enthalpies of pure components on the normalized depression at eutectic point is studied. The different curves correspond to different melting entropy values. In contrast to melting temperatures of pure components, the absolute values of the melting enthalpies of components affect the normalized depression at eutectic point. The lower the values of melting enthalpies, the higher the depression at eutectic point. As can be noticed from Figure 3, if the value of the melting enthalpy of Component 1 is high no depression is observed. In addition, only the low value of melting enthalpy (5 ${\mathrm{kJ}\;\mathrm{mol}}^{-1}$) of both components can affect the depression at eutectic point—this can be seen by the shift between solid and dashed lines, while the dotted and the dashed lines are the same. In conclusion, for ideal eutectic systems in which the components possess different melting temperatures, only low value of melting enthalpies of components can lead to deep eutectics.

Figure 4 shows how the eutectic composition depends on melting properties for the systems shown in Figure 2 and Figure 3. As can be seen in Figure 4, an equimolar eutectic composition is possible if both the melting enthalpies and melting temperatures of the components are equal. The eutectic composition is located very close to the pure Component 2 side (x_1,e_ = 0) if the difference between components melting temperatures or the melting enthalpy of Component 1 is of a high value. The low melting enthalpy value of Component 1 shifts the eutectic composition to a higher content of Component 1 (toward higher x_1,e_ values). The change in the absolute values of the melting enthalpies of components shifts the eutectic composition to different values. However, the effect of melting properties on the eutectic composition is lower compared to that on the depression at eutectic point—compare the shift between black and blues curves in Figure 2 and Figure 3 to that in Figure 4.

#### 3.1.2. Non-Ideality of Components in Liquid Phase

**Figure 5:**
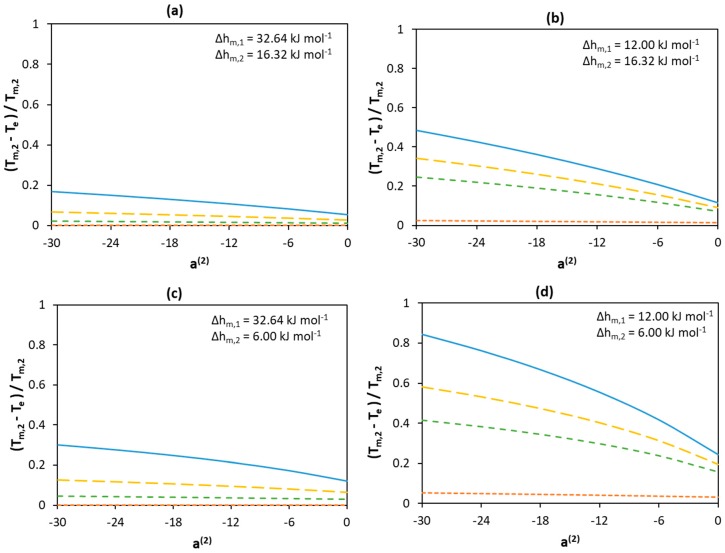
Effect of non-ideality of components on the normalized depression at eutectic temperature relative to the melting temperature of Component 2 assuming different melting enthalpy values of components (**a**) $\Delta h_{m,1}=32.64{\;\mathrm{kJ}\;\mathrm{mol}}^{-1}$ and $\Delta h_{m,2}=16.32{\;\mathrm{kJ}\;\mathrm{mol}}^{-1}$ (**b**) $\Delta h_{m,1}=12.00{\;\mathrm{kJ}\;\mathrm{mol}}^{-1}$ and $\Delta h_{m,2}=16.32{\;\mathrm{kJ}\;\mathrm{mol}}^{-1}$ (**c**) $\Delta h_{m,1}=32.64{\;\mathrm{kJ}\;\mathrm{mol}}^{-1}$ and $\Delta h_{m,2}=6.00{\;\mathrm{kJ}\;\mathrm{mol}}^{-1}$ (**d**) $\Delta h_{m,1}=12.00{\;\mathrm{kJ}\;\mathrm{mol}}^{-1}$ and $\Delta h_{m,2}=6.00{\;\mathrm{kJ}\;\mathrm{mol}}^{-1}$. The melting temperatures of Components 1 and 2 are set to 600 K and 300 K, respectively. The parameter value is only a measure for non-ideality with no physical significance. Legend: 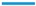 a^(1)^ = −30; 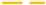 a^(1)^ = −18; 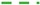 a^(1)^ = −12; 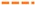 a^(1)^ = 0.

The effect of both parameters—namely the activity coefficients and melting properties—is demonstrated here for a hypothetical binary system. As shown in the previous section, the absolute values of the melting temperatures of components have no effect on the normalized depression at eutectic point at fixed melting entropies. Therefore, arbitrary melting temperatures of the high melting Component 1 and the low melting Component 2 are set to 600 K and 300 K, respectively. The melting enthalpy and entropy is used to investigate the effect of melting properties. Four cases are present in Figure 5. In the first case, the Walden rule (Equation (3)) is used to calculate the melting enthalpies. In the second case, the melting enthalpy of Component 1 (12.0 ${\mathrm{kJ}\;\mathrm{mol}}^{-1}$) is estimated assuming a low value of melting entropy of 20 ${J\;\mathrm{mol}}^{-1}\;K^{-1}$, whereas the melting enthalpy of Component 2 (16.32 ${\mathrm{kJ}\;\mathrm{mol}}^{-1}$) is calculated using the Walden rule. In the third case, the melting enthalpy of Component 2 (6.0 ${\mathrm{kJ}\;\mathrm{mol}}^{-1}$) is estimated using a low value of melting entropy of $20\;{J\;\mathrm{mol}}^{-1}\;K^{-1}$, whereas the melting enthalpy of Component 1 (32.64 ${\mathrm{kJ}\;\mathrm{mol}}^{-1}$) is calculated using the Walden rule. In the fourth case, the melting enthalpies of both components are estimated using a low value of melting entropy of $20\;{J\;\mathrm{mol}}^{-1}\;K^{-1}$. SLE are calculated using Equation (2) with activity coefficients screened using RK-polynomials (Equation (4)). The values of the parameters a^(i)^ in Equation (4) for each component are varied in the range −30–0 to account for the negative deviation from ideality ($\ln\left(\gamma_{i}^{L}\right)\;$< 0). It should be mentioned that these parameters have no significant physical meaning, and they are serving just as a measure for the value of the activity coefficients—i.e., the lower the value of parameter a^(i)^, the lower the value of $\gamma_{i}^{L}$ at the eutectic point.

Figure 5 demonstrates how solution non-ideality affects depression at the eutectic temperature for different melting enthalpies of the components. As in the case of the ideal mixture, the lower the melting enthalpy of Component 1, the larger the normalized depression at eutectic temperature. When the melting enthalpy of Component 1 is of a low value—see Figure 5b,d—depression of the melting point occurs, even if Component 1 behaves ideally (a^(1)^ = 0; orange curves in Figure 5b,d). However, when the melting enthalpy of Component 1 is of a high value—see Figure 5a,c—depression of the melting temperature is possible only if the activity coefficients of both components deviate considerably from unity—i.e., a^(1)^ < 0. Despite the fact that a low melting enthalpy value of Component 2 leads to an even more pronounced decrease in the eutectic temperature—as shown in Figure 5c,d—this occurs only if the activity coefficients of Component 1 are significantly lower than unity.

Generally, low component activity coefficients lead to a significant depression of the eutectic temperature of the mixture, independent of the melting enthalpy values. However, when the melting enthalpy of Component 1 is of a high value, only a small depression is observed when its activity coefficients are equal (or close) to unity (a^(1)^ = 0, orange curves in Figure 5a,c). In contrast, when Component 1 significantly deviates from ideality, a larger effect can be seen even if Component 2 behaves ideally (compare an orange curve with the lowest point of a blue curve at a^(2)^ = 0). In addition, comparing the change in eutectic temperature depression when a^(1)^ equals −30 and 0 at a^(2)^ = −30(the *y*-axis intercepts of the blue and orange curves) and when a^(2)^ equals −30 and 0 at a^(1)^ = −30 (the sides of the blue curves) indicates a weaker contribution of the non-ideality of Component 2 to decreasing the eutectic temperature (as compared with that of Component 1). This shows that compared with Component 2, the non-ideality of Component 1 is more significant in the formation of deeper eutectic mixtures.

**Figure 6:**
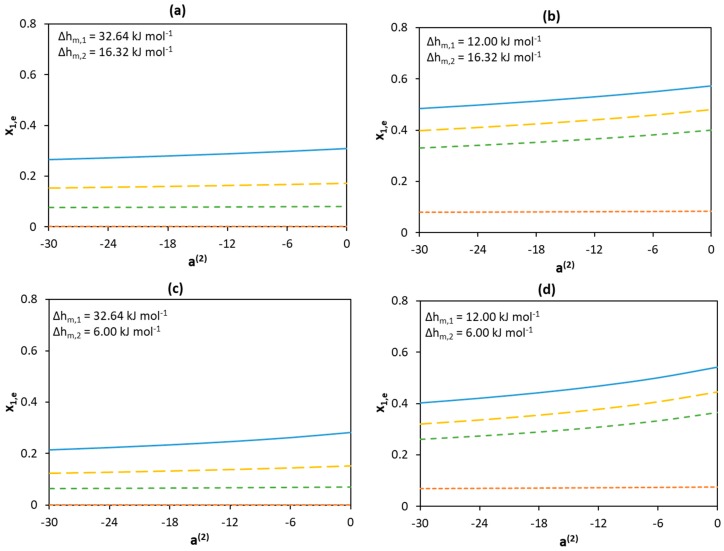
Effect of activity coefficients and melting enthalpies of the components on the eutectic mole fraction of Component 1 assuming different melting enthalpy values of components (**a**) $\Delta h_{m,1}=32.64{\;\mathrm{kJ}\;\mathrm{mol}}^{-1}$ and $\Delta h_{m,2}=16.32{\;\mathrm{kJ}\;\mathrm{mol}}^{-1}$ (**b**) $\Delta h_{m,1}=12.00{\;\mathrm{kJ}\;\mathrm{mol}}^{-1}$ and $\Delta h_{m,2}=16.32{\;\mathrm{kJ}\;\mathrm{mol}}^{-1}$ (**c**) $\Delta h_{m,1}=32.64{\;\mathrm{kJ}\;\mathrm{mol}}^{-1}$ and $\Delta h_{m,2}=6.00{\;\mathrm{kJ}\;\mathrm{mol}}^{-1}$ (**d**) $\Delta h_{m,1}=12.00{\;\mathrm{kJ}\;\mathrm{mol}}^{-1}$ and $\Delta h_{m,2}=6.00{\;\mathrm{kJ}\;\mathrm{mol}}^{-1}$. The melting temperatures of Components 1 and 2 are set to 600 K and 300 K, respectively. The parameter value is only a measure for non-ideality with no physical significance. Legend: 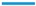 a^(1)^ = −30; 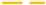 a^(1)^ = −18; 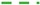 a^(1)^ = −12; 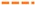 a^(1)^ = 0.

Figure 6 shows the effect of the non-ideality of the liquid phase on eutectic composition at different melting enthalpies. The low activity coefficients of Component 1 significantly shift the eutectic point to a higher content of Component 1 (toward higher x_1,e_ values), whereas an opposite effect is observed for the low activity coefficients of Component 2 (toward lower x_1,e_ values). However, the effect of the activity coefficients of Component 1 is significantly larger than that of Component 2 (see Figure 5c).

To summarize, the melting properties as well as the activity coefficients of the components can affect the position of the eutectic point. Eutectic composition shifts to the side of the component that has lower activity coefficients as well as a lower melting enthalpy. If both components have similar activity coefficients and melting enthalpies, the eutectic composition might occur at a molar ratio of 1:1. Low melting enthalpies and activity coefficients of both components lead to the greatest depression of the eutectic temperature of the mixture. The melting properties and non-ideality of the high melting component have a more pronounced effect on eutectic temperature depression. Therefore, the search for new deep eutectic mixtures should likely be based on the identification of compounds with low melting enthalpy, especially if they also possess a high melting temperature.

### 3.2. Parameter Study on Real Binary Systems

#### 3.2.1. [Ch]Cl-Based Binary Systems

Choline chloride ([Ch]Cl) is a common constituent (HBA) of DESs. The melting properties of [Ch]Cl are not available experimentally owing to its thermal instability. The decomposition temperature of [Ch]Cl is around 575 K. Recently, Fernandez et al. [56] made an indirect assessment of the melting properties of [Ch]Cl. Experimental SLE data in binary mixtures of [Ch]Cl with different ILs were fitted to the ideal solution model, and the [Ch]Cl melting properties were calculated correspondingly. The values obtained were melting enthalpy = 4.3 ${\mathrm{kJ}\;\mathrm{mol}}^{-1}$ and melting temperature = 597 K.

**Figure 7:**
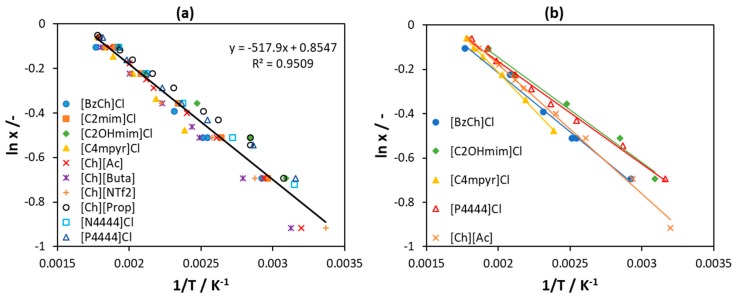
(**a**) Solubility of choline chloride ([Ch]Cl) in several ILs regressed to a linear function. (**b**) Solubility of [Ch]Cl in selected ILs, each regressed to a linear function. Data are taken from [56].

According to Equation (2) applied to an ideal solution ($\gamma_{i}^{L}=1$), the values of ln(x_1_) plotted as a function of the reciprocal temperature (1/T) should fit a straight line with the *y*-axis intercept equal to $\left(\Delta h_{m,1}/\left({\mathrm{RT}}_{m,1}\right)\right)$ and the slope equal to $(-\Delta h_{m,1}/R$). The corresponding plot for 10 [Ch]Cl/IL systems used for fitting the melting properties of [Ch]Cl is shown in Figure 7a. The points represent the experimental data for all 10 [Ch]Cl/IL systems, and the line is the fitted straight line used for the calculation of the [Ch]Cl melting properties given above. As can be seen from Figure 7a, the value of determination coefficient (R^2^) indicates a rather good linear regression. However, the points for the individual binaries are systematically distributed to the sides of the fit. For example, all points for [C_2_OHmim]Cl lie above the fit, whereas those for [BzCh]Cl lie below the fit. It is worth noting that the *y*-axis shows logarithmic values and the *x*-axis reciprocal values; therefore, a small shift from the fitted line corresponds to a significant difference in the calculated T and x. As shown in Figure 7b, the lines fitted to five single binaries [Ch]Cl/IL systems have different slopes and intercepts; therefore, they lead to different [Ch]Cl melting properties. The melting temperature is in the range 588.9–630.8 K and melting enthalpy is in the range 3.85–5.63 kJ mol^−1^, being within around 7% and 40% from the mean value, respectively. See Table S1 in supplementary material for detailed results.

**Figure 8:**
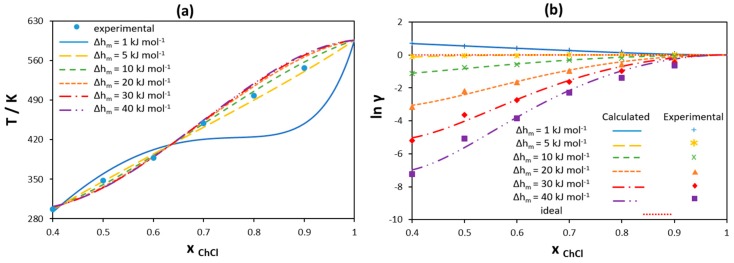
(**a**) Liquidus line of [Ch]Cl in a [Ch]Cl/choline acetate binary mixture modeled using the RK-polynomial assuming different melting enthalpy values. (**b**) Activity coefficients of [Ch]Cl in the liquid phase calculated using the RK-polynomial assuming different melting enthalpy values. Melting temperature of [Ch]Cl is assumed to be 597 K. Experimental data are taken from [56].

The ideal solution model accounts for the melting properties of one component only, ignoring any dissimilarity of the second components in the mixture, which can be described by activity coefficients. In order to analyze the possible non-ideality of [Ch]Cl in the liquid phase, SLE of binary mixtures of [Ch]Cl and ILs have been modeled assuming different values of [Ch]Cl melting enthalpy using the RK-polynomial with two parameters to estimate the activity coefficients. The values of [Ch]Cl melting temperature estimated from single [Ch]Cl/IL mixtures scatter within 7% only, and as shown in Section 3.1.1 for the hypothetical ideal system, the absolute values of melting temperatures have on effect on the eutectic point properties. Hence, the value of 597 K estimated by Fernandes et al. [56] has been used in the present work. Figure 8a shows the obtained results for the [Ch]Cl liquidus line in the [Ch]Cl/choline acetate [Ch][Ac] binary system as an example. Figure 8b represents the corresponding data for activity coefficients of [Ch]Cl, wherein the points correspond to the values calculated from the experimental data using Equation (2) and the lines give the best fit using the RK-polynomial. As shown in Figure 8a, it is possible to model the SLE data with rather good agreement assuming different values of [Ch]Cl melting enthalpy, except in the cases of very low (1 kJ mol^−1^) values. However, as seen from Figure 8b, the experimental activity coefficients of [Ch]Cl in the liquid phase change as the melting enthalpy of [Ch]Cl changes. This is because a variation in the melting enthalpy indicates a change in the value of the reference state chemical potential to which the activity coefficients refer. [Ch]Cl shows ideal behavior when the melting enthalpy has a low value of 5 ${\mathrm{kJ}\;\mathrm{mol}}^{-1}$, a value close to that found by Fernandez et al. [56].

**Figure 9:**
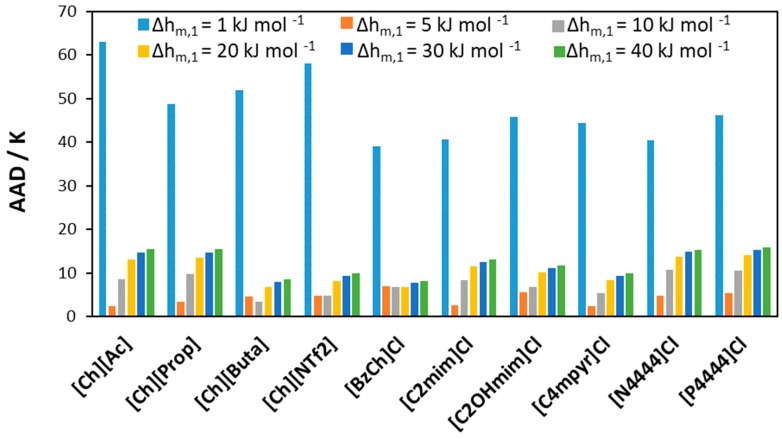
AAD between calculated and experimental liquidus temperatures for binary mixtures of [Ch]Cl and ILs assuming different melting enthalpies for [Ch]Cl.

Results for other studied [Ch]Cl/IL systems can be found in Figures S1–S3 in the supplementary material. Figure 9 shows the absolute average deviation (AAD) obtained when screening the melting enthalpy of [Ch]Cl in all studied [Ch]Cl/IL binary mixtures. The high error in modeling the solubility of [Ch]Cl using a very low melting enthalpy (1 kJ mol^−1^) indicates that such a value might be unreasonable. At the same time, experimental data can be modeled with low AAD when the value of Δh_m,1_ is in the range 5–40 kJ mol^−1^.

**Figure 10:**
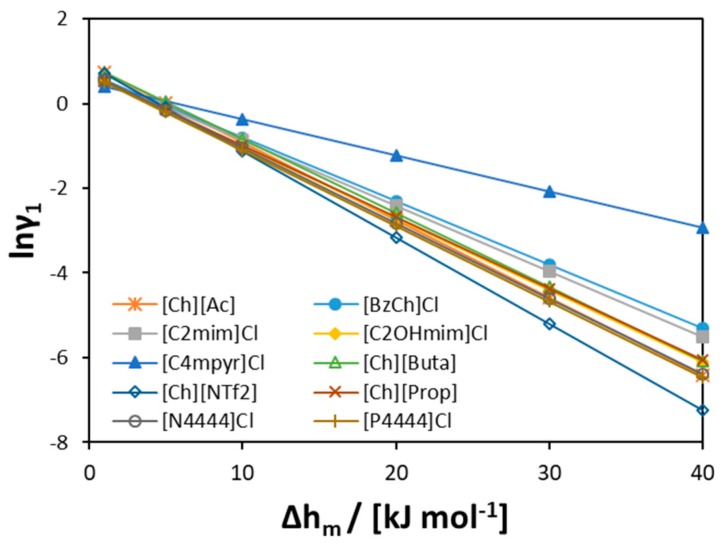
The activity coefficients of [Ch]Cl at the experimental eutectic point estimated assuming different melting enthalpies of [Ch]Cl.

**Figure 11:**
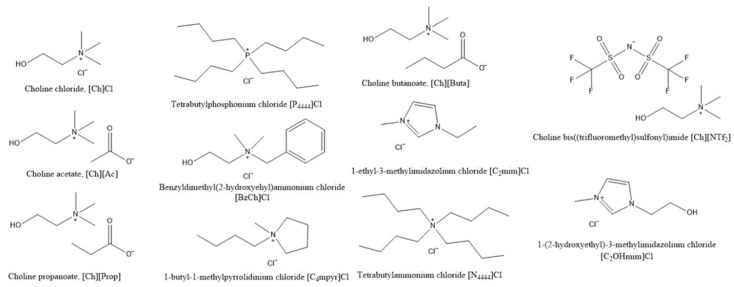
Chemical names and structures of the [Ch]Cl and ILs studied.

To further explore [Ch]Cl behavior in the liquid phase, the value of the activity coefficients of [Ch]Cl at the experimental eutectic point can be analyzed (see Figure 10). As seen from the figure, at low melting enthalpy values of [Ch]Cl (<5 kJ mol^−1^), the activity coefficients are close to one in all previously studied binary systems (as found by Fernandez et al. [56]). However, at higher [Ch]Cl melting enthalpies (>20 kJ mol^−1^), the values of the activity coefficients change (see Figure 10). As seen in Figure 11, the molecular structures of ILs significantly differ from each other and from [Ch]Cl, that would result in different hydrophobicities. Owing to the long alkyl chains in [P4444]^+^ and [N4444]^+^ cations, these cations are more hydrophobic than the [Ch]^+^ cation; hence, the interactions in solution would differ from those of the pure components. At low values of [Ch]Cl melting enthalpy, the difference in the interactions is concealed, leading to quasi-ideal behavior. However, at higher values, the difference in molecular size and hydrophobicity appears obvious.

Despite the fact that predicting [Ch]Cl solubility in ILs is possible by assuming an ideal solubility model with a low melting enthalpy of [Ch]Cl, system-specific information such as the interactions of [Ch]Cl with different ILs can be hidden by the system ideality assumption. Martins et al. [49] also suggested that the deep depression of the eutectic temperature in [Ch]Cl-based mixtures is due to the low value of its melting enthalpy. Assessing these important features of such systems is an advantage of modeling the non-ideality in the liquid phase. An apparent changing of the melting enthalpies of the components leads to different activity coefficients in the liquid phase. This leads to a different conclusion about the intermolecular interactions and the behavior of the components in the system, which might be beneficial in decreasing the complexity of SLE modeling. However, a discussion of SLE results might be impractical based on this empirical approach.

#### 3.2.2. Quaternary Ammonium Chloride Salts/Fatty Acids DES

The SLE of binary mixtures of different quaternary ammonium chlorides and fatty acids have been studied in detail [46]. These systems are considered DESs owing to strong negative deviation from ideality in quaternary ammonium chloride liquidus lines. Pontes et al. [46] used PC-SAFT to model liquid phase non-ideality. The modeling results are in good agreement with experimental data. The melting properties of these salts reported in Pontes et al. [46] are summarized in supplementary materials. Pontes et al. [46] confirmed the decomposition of [N_1111_]Cl, and its melting properties were estimated using SLE data and activity coefficients calculated using COSMO-RS. For the other two salts namely, [N_2222_]Cl and [N_3333_]Cl, the melting properties were measured in Büchi apparatus and DSC. However, in earlier literature, these salts were reported to decompose at temperatures below their melting temperatures [57,58]. As a result of decomposition, the melting enthalpy measured by DSC for the salts might represent the heat of decomposition and not the melting enthalpy of these salts.

**Figure 12:**
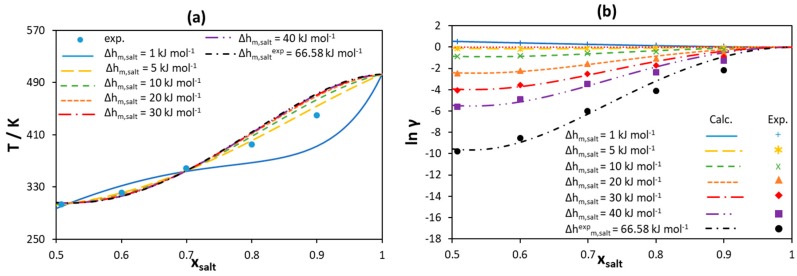
(**a**) [N_3333_]Cl liquidus line for a [N_3333_]Cl/palmitic acid binary mixture modeled using the Redlich–Kister polynomial with three parameters assuming different melting enthalpy values. (**b**) Activity coefficients of [N_3333_]Cl in the liquid phase calculated using the Redlich–Kister polynomial assuming different melting enthalpy values. Experimental data taken from [46].

In this work, the different melting enthalpy values of quaternary ammonium chloride are screened and the activity coefficients are modeled using the Redlich–Kister polynomial with three parameters. The [N_3333_]Cl/palmitic acid system shows the strongest negative deviation from ideality compared with the other systems studied in Pontes et al. [46]; therefore, its SLE data are analyzed here in detail (see Figure 12). Results for the other systems can be found in Figures S5–S7 in supplementary material.

As can be seen in Figure 12a, the liquidus lines of [N_3333_]Cl estimated using the RK-polynomials are in good agreement with experimental data for any melting enthalpy assumed, except for the very low melting enthalpy (1 kJ mol^−1^). As for [Ch]Cl/IL systems, the melting enthalpy of 5 kJ mol^−1^ leads to ideal solubility for [N_3333_]Cl (see the yellow curve in Figure 12b). However, the larger the value of melting enthalpy, the stronger the negative deviations are from ideality required to reproduce the experimental data. Therefore, assuming a very high melting enthalpy would overestimate the non-ideality, making it rather difficult to account for using well-established and relatively simple-to-use activity coefficient models.

Table 1 shows the eutectic point properties for all quaternary ammonium chloride/fatty acid binary mixtures studied in [46]. Along with the experimental eutectic composition and temperature (x_e,acid_ and T_e_), the table shows the normalized difference between the eutectic temperature predicted by PC-SAFT in [46] and the melting temperature of the acid (ΔT_e_). As can be seen from Table 1, the largest depression in melting temperature of the mixture at the eutectic point relative to the melting temperature of the acid is for [N_3333_]Cl/palmitic acid system (ΔT_e_ = 0.1096). In addition, the [N_3333_]Cl/palmitic acid system has the eutectic composition with the highest salt mole fraction, as compared with any other quaternary ammonium chloride/fatty acid systems (x_e,salt_ = 0.517). This seems to be due to the strong interactions between [N_3333_]Cl and palmitic acid, as was shown in [46] (see also the parameter study section for the link between activity coefficients and eutectic composition). As seen from Table 1, ΔT_e_ values show that quaternary ammonium chlorides/ fatty acids DESs do not show deep eutectic behavior based on the normalized depression at eutectic point. However, based on the definition of Kollau et al. [47] and Martins et al. [49] for deep eutectic solvents, these systems would be considered as deep eutectic systems. The small depression at eutectic temperature relative to the acid melting temperature might be a result of the high melting enthalpies of the fatty acids. As shown in the parameter study, melting properties of components affect the depression at eutectic point in a larger extent compared to the eutectic composition. In contrast, activity coefficient values significantly shift the eutectic composition to the side of the component with low activity coefficients. Therefore, the eutectic composition gives an indication for the component with the larger negative deviation from ideality.

To summarize, changing the melting enthalpy value of quaternary ammonium chloride changes the activity coefficient values and thus hides or increases the actual complexity of the systems. Eutectic systems with a high deviation from ideality do not always have a large depression at eutectic temperature; rather, they would have a large shift in the eutectic composition away from pure components sides.

## 4. Method

To study the effect of melting properties and intermolecular interactions on eutectic point properties, a hypothetical binary system is assumed. The system is considered to be either ideal ($\gamma_{i}^{L}=1$) in order to only study the effect of melting properties, or non-ideal (with negative deviations from ideality; $\gamma_{i}^{L}<1$) to consider the effect of intermolecular interactions. Different values of melting enthalpy and melting temperature are screened for each of the components. In some cases, the melting enthalpy values have been estimated using the Walden rule. The Walden rule states that the melting entropy of rigid molecules is constant, with a value of 54.4 ${J\;\mathrm{mol}}^{-1}K^{-1}$ [52]. The melting enthalpy can thus be calculated as

(3) $$\Delta h_{m}=54.4\;T_{m}$$

To model the non-ideality of components, the activity coefficients of components in the liquid phase are calculated using Redlich–Kister (RK) polynomial [51]

(4) $$RT\ln\gamma_{i}=a^{\left(i\right)}x_{j}^{2}+b^{\left(i\right)}x_{j}^{3}+c^{\left(i\right)}x_{j}^{4}\ldots .$$

When the activity coefficients are screened (Section 3.1.2), only the first term with one parameter is used. In this case, when parameter $a^{\left(i\right)}$ is equal to zero, the ideal case is described; whereas $a^{\left(i\right)}<0$ describes favored interactions between unlike molecules in the liquid phase—i.e., negative deviation from ideality. Positive deviation from ideality leads to a higher liquidus temperature compared with the ideal case; therefore, values of parameter $a^{\left(i\right)}>0$ are not considered in this work. The parameters are empirical and do not provide any physical significance. They are just used as a measure of different values of activity coefficients of components.

For modeling the non-ideality in [Ch]Cl/ILs binary systems (Section 3.2.1), two terms with two parameters are used. Binary mixtures of quaternary ammonium chlorides with fatty acids (Section 3.2.2) are more complex; hence, three terms with three parameters are used. In the latter two cases, for a given melting enthalpy value, the RK-parameters are fitted to the SLE experimental data by minimizing the objective function

(5) $$OF=\sum {\left(\gamma_{1}^{cal}-\gamma_{1}^{exp}\right)}^{2}$$

where $\gamma_{1}^{cal}$ is the calculated activity coefficient of Component 1 in the liquid phase using the RK-polynomial and $\gamma_{1}^{exp}$ is the activity coefficient of Component 1 in the liquid phase estimated using Equation (2) and measured SLE taken from the literature.

To evaluate the quality of the calculated SLE data, the AAD between the calculated ($T_{i}^{cal})$ and experimental ($T_{i}^{exp})$ liquidus temperatures is calculated as

(6) $$AAD=\frac{1}{N}\overset{N}{\underset{i}{\sum }}\left|T_{i}^{cal}-T_{i}^{exp}\right|$$

## 5. Conclusions

The parameter study carried out in the present work shows that (i) obtaining the accurate melting properties of pure constituents and (ii) adequate modeling of non-ideality in the liquid phase are essential for prediction of the SLE in DESs. Deep eutectics can be a result of low melting enthalpies, low activity coefficients, or both. When analyzing the modeled SLE diagrams, the estimate of eutectic composition can provide a better indication of system non-ideality. Components with lower activity coefficient values have a higher mole fraction at the eutectic point. In systems of two components with similar melting enthalpies and similar activity coefficients, the eutectic composition is observed at around a 1:1 molar ratio of the two components. Therefore, designing DES systems should simultaneously take into account melting properties as well as interactions between unlike molecules. However, strong negative deviation from ideality is not the only reason for DES formation; substances with low melting enthalpies could also form rather deep eutectics.

Recent work on the SLE of [Ch]Cl-based DES systems has reported that under an assumption of a low melting enthalpy value (4.3 kJ mol^−1^), [Ch]Cl behaves quasi-ideally in most such systems. However, molecular experiments and simulations in the recent literature have revealed that [Ch]Cl-based systems behave in a complex manner. As demonstrated in the present study, the solubility of [Ch]Cl in different ILs can be adequately modeled assuming different melting enthalpy values. Small values lead to a nearly ideal behavior in the liquid phase, whereas it is necessary to account for activity coefficients at higher melting enthalpy values. Owing to the absence of any experimental data on the melting enthalpy of [Ch]Cl for comparison, it is not possible to determine which of the values is true. However, owing to the differences in size and hydrophobicity of the studied ILs, differences in their interactions with [Ch]Cl should be expected. Thus, the low melting enthalpy value may conceal the complexity of interactions in the liquid phase and should be used with caution. Therefore, modeling the SLE of [Ch]Cl-based DESs remains unclear; more research is necessary to fully understand the behavior of these systems.

For DES systems with strong intermolecular interactions between unlike molecules, the depression in melting temperature at the eutectic point is not related only to activity coefficient values. The studied quaternary ammonium chloride/fatty acid systems show strong negative deviation from ideality with small depression at eutectic point relative to the acid melting temperature. At the same time, the systems show a shift in the eutectic composition to the side of the salt. Therefore, the eutectic composition can provide an indication of strong interactions—e.g., hydrogen bonds—between DES components.

From the application viewpoint when designing a DES, no strict definition of a DES system is necessary; rather, a better understanding of the system behavior and intermolecular interactions, reliable data for the SLE of mixtures and pure components, and an assessment of system suitability for an actual application are required. In fact, any simple eutectic mixture with a large depression in eutectic temperature relative to the melting temperatures of the pure constituents sufficient to form a liquid at operating temperature could be referred to as a DES (or NADES). In searching for new DESs, high melting compounds with low melting enthalpies—which have strong interactions with the second component in the liquid mixture but not in its pure solid state—should be sought. The non-ideality of the liquid phase can thereby be directly estimated using well-developed and easy-to-use thermodynamic tools such as activity coefficient models and equations of state.

## Figures and Tables

**Table 1:** Comparison of eutectic composition, eutectic temperature, and the normalized difference between the eutectic temperature estimated using PC-SAFT and the melting temperature of the acids. Data are taken from [46].

	[N_1111_]Cl			[N_2222_]Cl			[N_3333_]Cl		
	x_e,acid_	T_e_	ΔT_e_	x_e,acid_	T_e_	ΔT_e_	x_e,acid_	T_e_	ΔT_e_
Capric acid	0.808	299.91	0.0161	0.614	297.40	0.0247	0.710	288.46	0.0565
Lauric acid	0.630	303.01	0.0478	0.640	301.44	0.0532	0.644	297.05	0.0688
Myristic acid	0.712	321.65	0.0167	0.658	317.88	0.0288	0.571	304.54	0.0738
Palmitic acid	0.615	328.51	0.0254	0.607	318.78	0.0567	0.483	303.56	0.1096
Stearic acid	0.634	337.89	0.0171	0.556	317.02	0.0841	0.562	322.82	0.0646

## Supplementary Materials

The following are available online at https://www.mdpi.com/1420-3049/24/12/2334/s1. Figure S1: Liquidus line and activity coefficients in the liquid phase of [Ch]Cl in a binary mixture of [Ch]Cl and (a) [Ch][Ac], (b)[Ch][Prop], and (c)[Ch][Buta], modeled using Redlich–Kister polynomial. Figure S2: Liquidus line and activity coefficients in the liquid phase of [Ch]Cl in a binary mixture of [Ch]Cl and (a) [Ch][NTf_2_], (b) [BzCh]Cl, (c) [C_2_ mim]Cl, and (d) [C_2_OHmim]Cl, modeled using Redlich–Kister polynomial. Figure S3: Liquidus line and activity coefficients in the liquid phase of [Ch]Cl in a binary mixture of [Ch]Cl and (a) [C_4_ mpyr]Cl, (b) [N_4444_]Cl, and (c) [P_4444_]Cl, modeled using Redlich–Kister polynomial. Figure S4: Chemical names and structures of [Ch]Cl and ILs studied. Figure S5: Liquidus line and activity coefficients of [N_1111_]Cl in a binary mixture of [N_1111_]Cl and (a) capric acid, (b) lauric acid, (c) myristic acid, (d) palmitic acid, and (e) stearic acid, modeled using the Redlich–Kister polynomial with three parameters assuming different melting enthalpy values. Figure S6: Liquidus line and activity coefficients of [N_2222_]Cl in a binary mixture of [N_2222_]Cl and (a) capric acid, (b) lauric acid, (c) myristic acid, (d) palmitic acid, and (e) stearic acid, modeled using the Redlich–Kister polynomial with three parameters assuming different melting enthalpy values. Figure S7: Liquidus line and activity coefficients of [N_3333_]Cl in a binary mixture of [N_3333_]Cl and (a) capric acid, (b) lauric acid, (c) myristic acid, (d) palmitic acid, and (e) stearic acid, modeled using the Redlich–Kister polynomial with three parameters assuming different melting enthalpy values. Table S1: Melting properties of [Ch]Cl obtained from linear regression of the solubility of [Ch]Cl in different ILs. Table S2: Empirical parameters of RK-polynomial for calculating activity coefficients of salts in binary mixtures of quaternary ammonium chloride salts and capric acid. Table S3: Empirical parameters of RK-polynomial for calculating activity coefficients of salts in binary mixtures of quaternary ammonium chloride salts and lauric acid. Table S4: Empirical parameters of RK-polynomial for calculating activity coefficients of salts in binary mixtures of quaternary ammonium chloride salts and myristic acid. Table S5: Empirical parameters of RK-polynomial for calculating activity coefficients of salts in binary mixtures of quaternary ammonium chloride salts and palmitic acid. Table S6: Empirical parameters of RK-polynomial for calculating activity coefficients of salts in binary mixtures of quaternary ammonium chloride salts and stearic acid. Table S7: Melting properties of quaternary ammonium chloride and fatty acids.
