# Supplementary material for: Modeling of Solid–Liquid Equilibria in Deep Eutectic Solvents: A Parameter Study
Source: Molecules. 2019 Jun 25;24(12):2334. doi: 10.3390/molecules24122334 (PMC6631263; doi:10.3390/molecules24122334)
Supplement: Supplementary file 1 [file molecules-24-02334-s001.zip › supplementary/Alhadid_SLE_parameter_study_supplementary.docx]

# Supplementary Material

Modeling of Solid-liquid Equilibria in Deep Eutectic Solvents: A Parameter Study

Ahmad Alhadid ^1^, Liudmila Mokrushina ^2^ and Mirjana Minceva ^1,^*

^1^ Technical University of Munich, Biothermodynamics, TUM School of Life and Food Sciences Weihenstephan, Maximus-von-Imhof-Forum 2, 85354 Freising, Germany; [ahmad.alhadid@tum.de](mailto:ahmad.alhadid@tum.de); mirjana.minceva@tum.de

^2^ Friedrich-Alexander-Universität Erlangen-Nürnberg (FAU), Separation Science & Technology, Egerlandstr. 3, 91058 Erlangen, Germany; liudmila.mokrushina@fau.de.

***** Correspondence: mirjana.minceva@tum.de


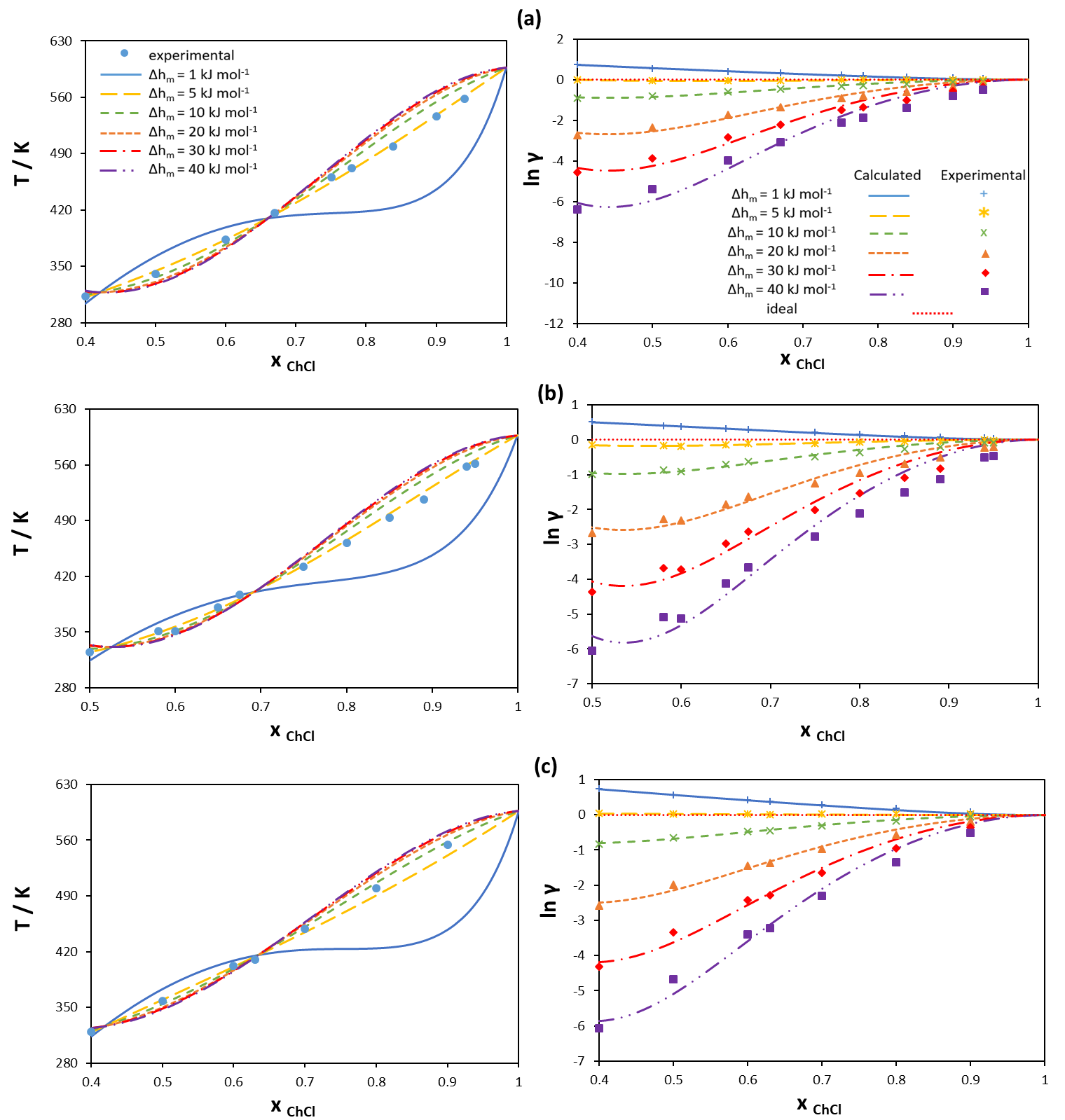


**Figure S1**. Liquidus line (left) and activity coefficients in liquid phase (right) of [Ch]Cl in binary mixture of [Ch]Cl and (a) [Ch][Ac] (b)[Ch][Prop] (c)[Ch][Buta] modeled using Redlich–Kister polynomial. Experimental data are taken from Fernandez et al. [1].


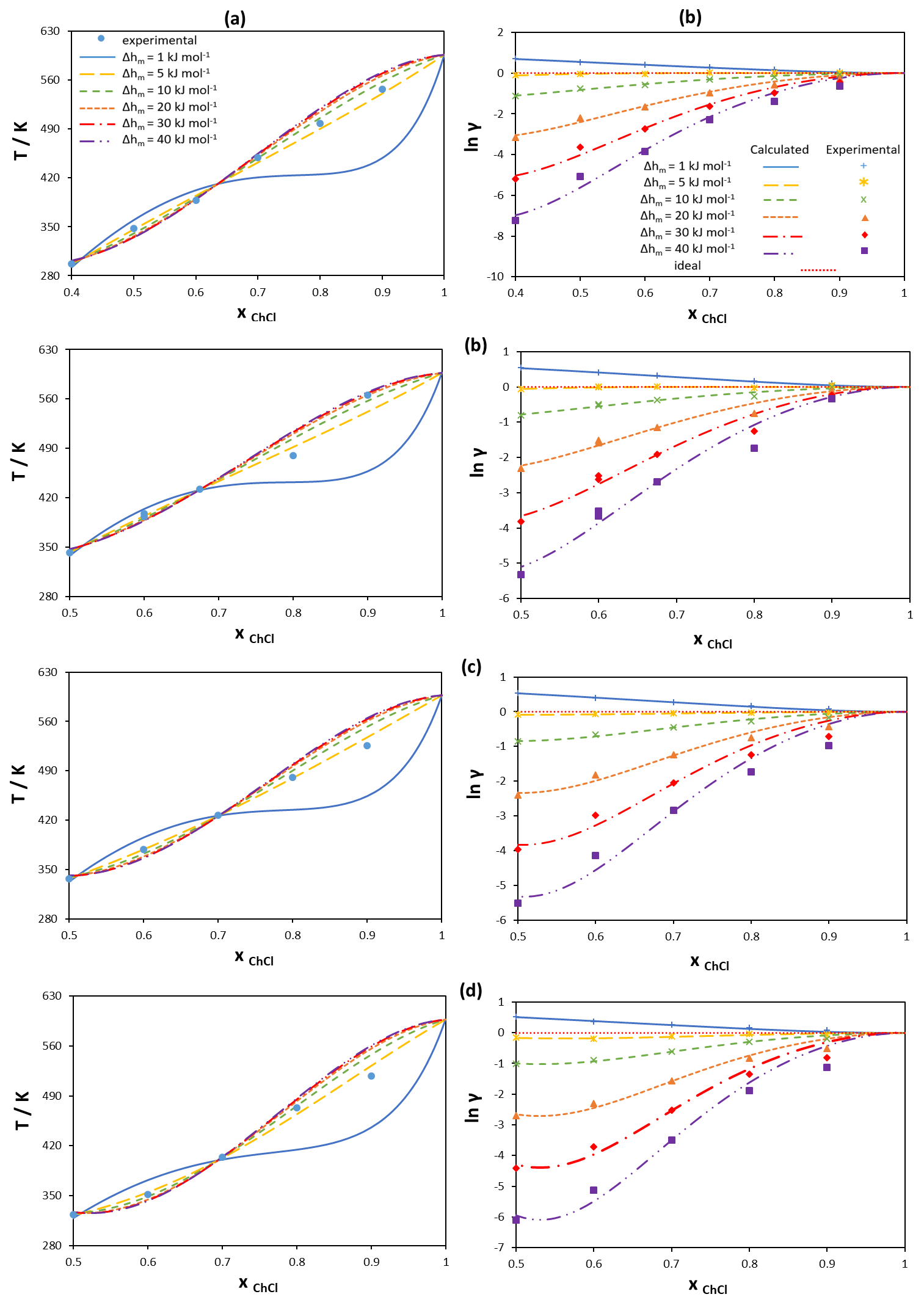


**Figure S2.** Liquidus line (left) and activity coefficients in liquid phase (right) of [Ch]Cl in binary mixture of [Ch]Cl and (a) [Ch][NTf_2_] (b) [BzCh]Cl (c) [C_2_mim]Cl (d) [C_2_OHmim]Cl modeled using Redlich–Kister polynomial. Experimental data are taken from Fernandez et al. [1].


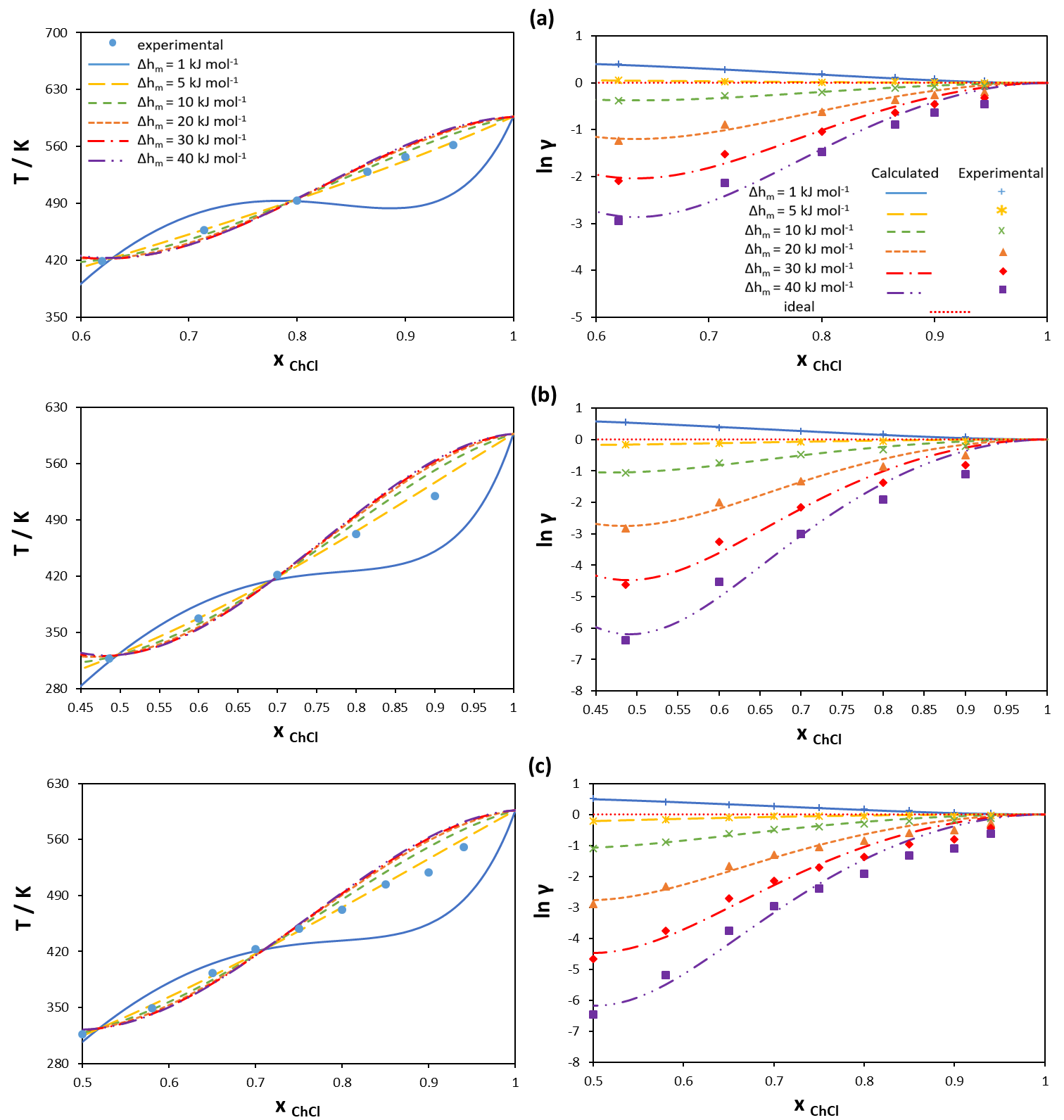


**Figure S3**. Liquidus line (left) and activity coefficients in liquid phase (right) of [Ch]Cl in binary mixture of [Ch]Cl and (a) [C_4_mpyr]Cl (b) [N_4444_]Cl (c) [P_4444_]Cl modeled using the Wilson equation. Experimental data are taken from Fernandez et al. [1].


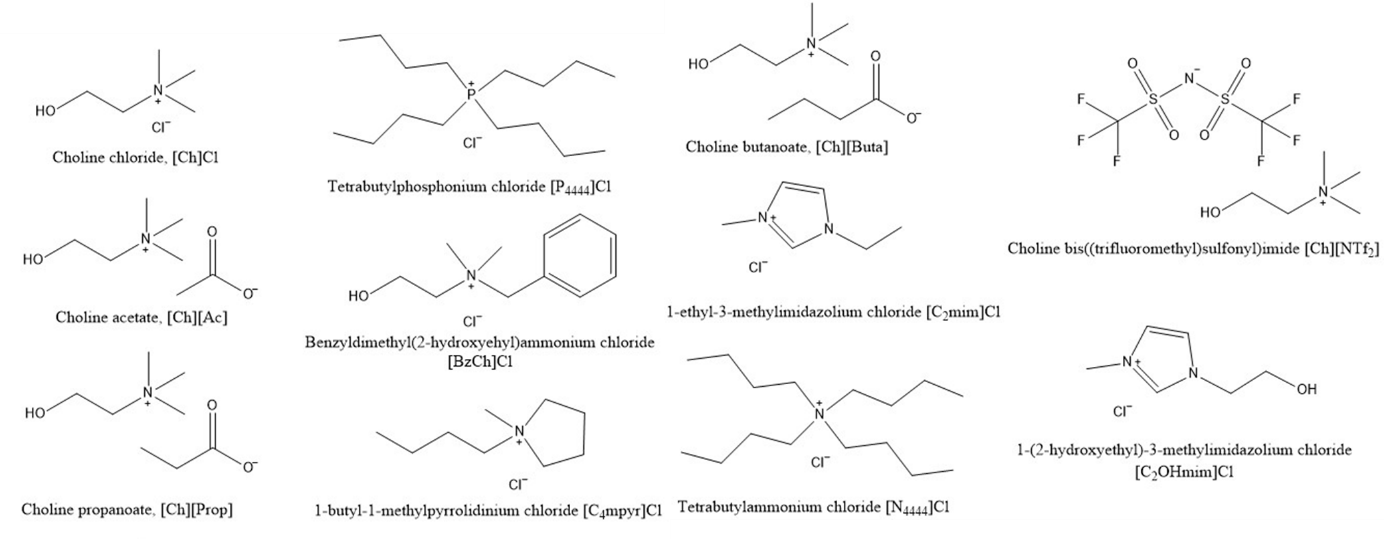


**Figure S4.** Chemical names and structures of [Ch]Cl and ILs studied.

T**able S1**. Melting properties of [Ch]Cl obtained from linear regression of solubility of [Ch]Cl in different ILs.

| IL | T_m_ / K | Δh_m_ / kJ mol^-1^ |
| --- | --- | --- |
| [Ch][Ac] | 588.92 | 4.86 |
| [Ch][Prop] | 594.39 | 3.90 |
| [Ch][Buta] | 607.94 | 5.06 |
| [Ch][NTf_2_] | 630.83 | 4.34 |
| [BzCh]Cl | 628.26 | 4.41 |
| [C_2_mim]Cl | 591.61 | 4.52 |
| [C_2_OHmim]Cl | 589.51 | 3.95 |
| [C_4_mpyr]Cl | 593.32 | 5.63 |
| [N_4444_]Cl | 594.22 | 4.11 |
| [P_4444_]Cl | 607.16 | 3.85 |
| Average | 602.61 | 4.46 |


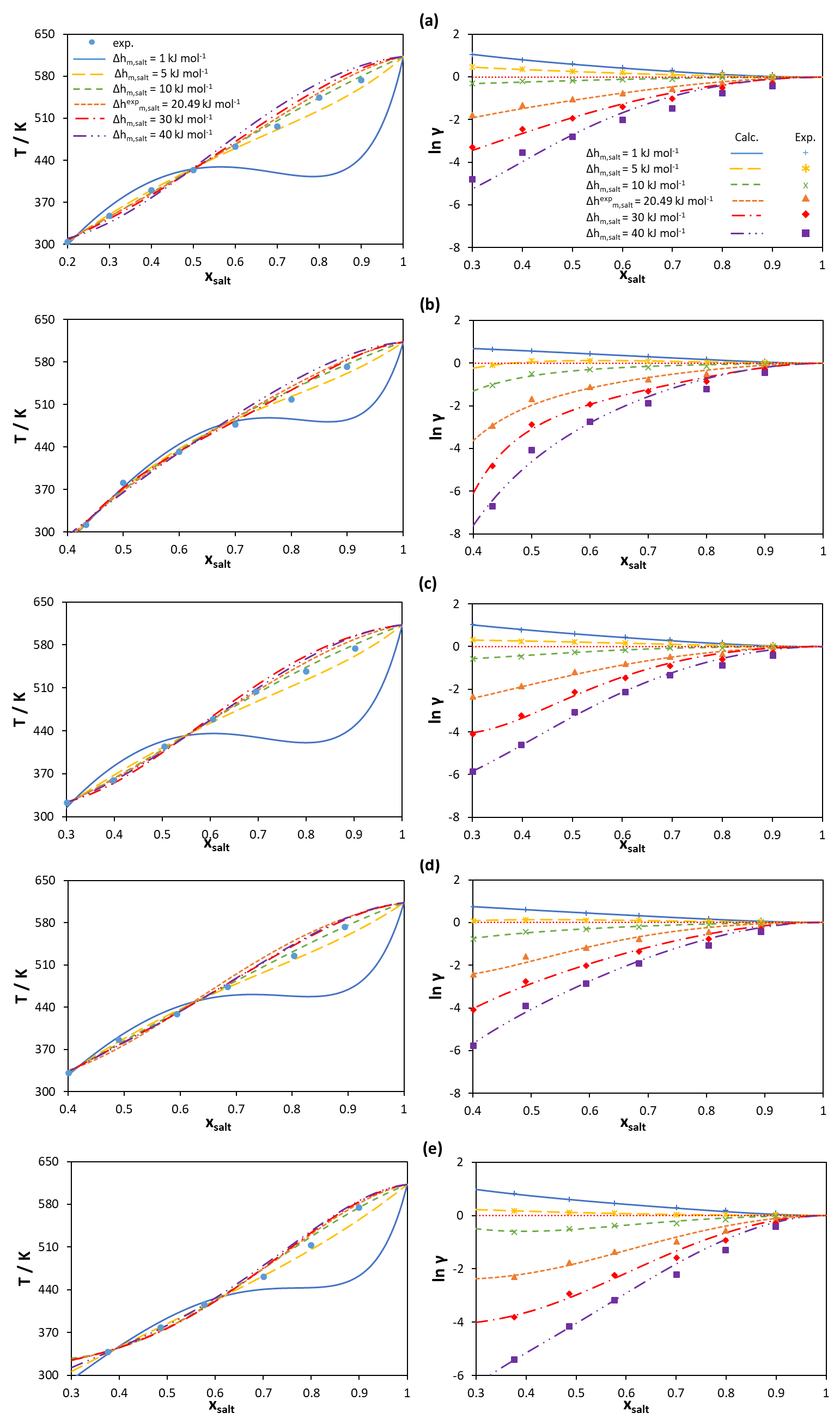


**Figure S5.** Liquidus line (left) and activity coefficients (right) of [N_1111_]Cl in binary mixture of [N_1111_]Cl and (a) capric acid (b) lauric acid (c) myristic acid (d) palmitic acid (e) stearic acid modeled using Redlich-Kister polynomial with three parameters assuming different melting enthalpy values. Experimental data are taken from [2].


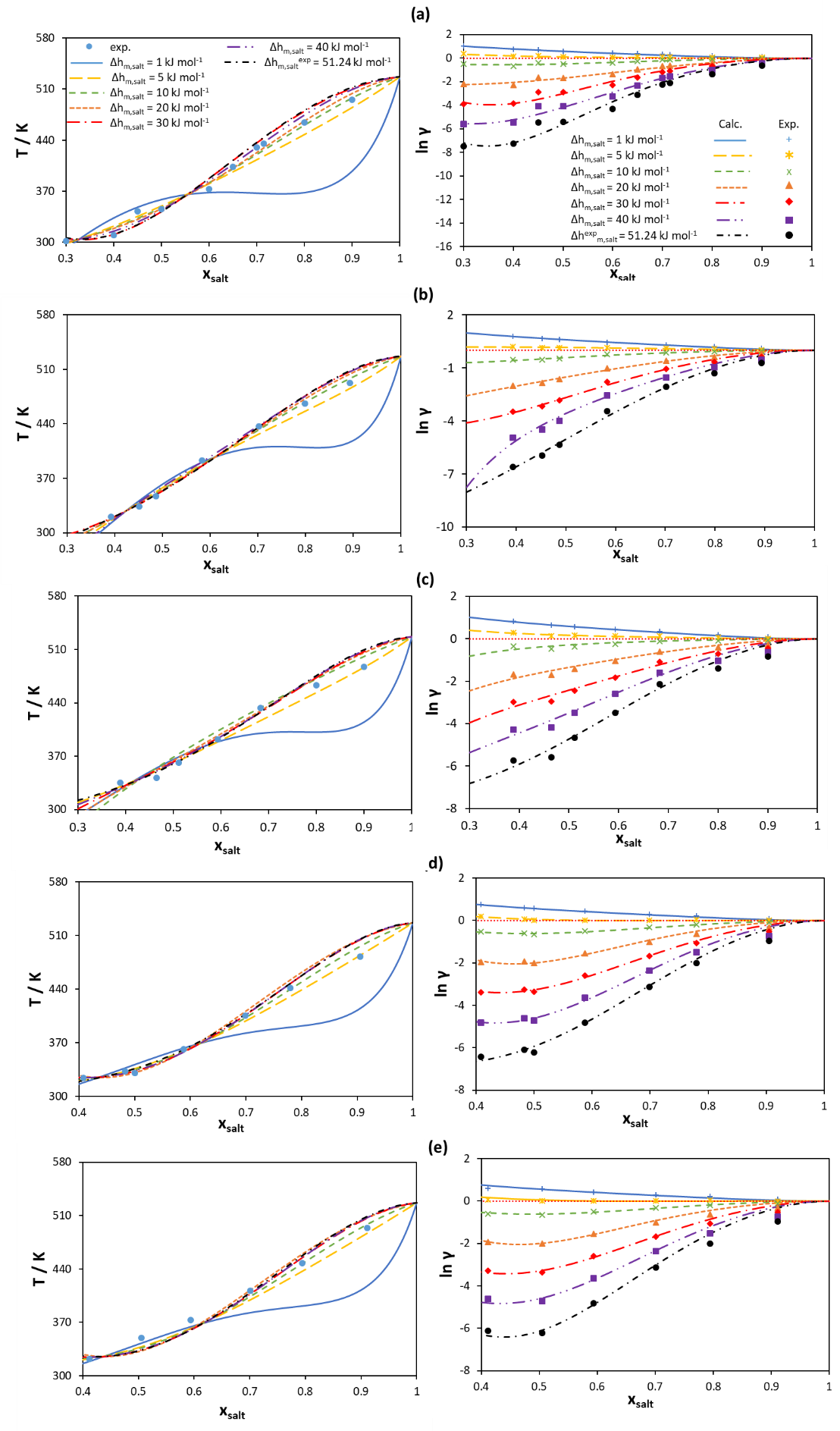


**Figure S6**. Liquidus line (left) and activity coefficients (right) of [N_2222_]Cl in binary mixture of [N_2222_]Cl and (a) capric acid (b) lauric acid (c) myristic acid (d) palmitic acid (e) stearic acid modeled using Redlich-Kister polynomial with three parameters assuming different melting enthalpy values. Experimental data are taken from [2].


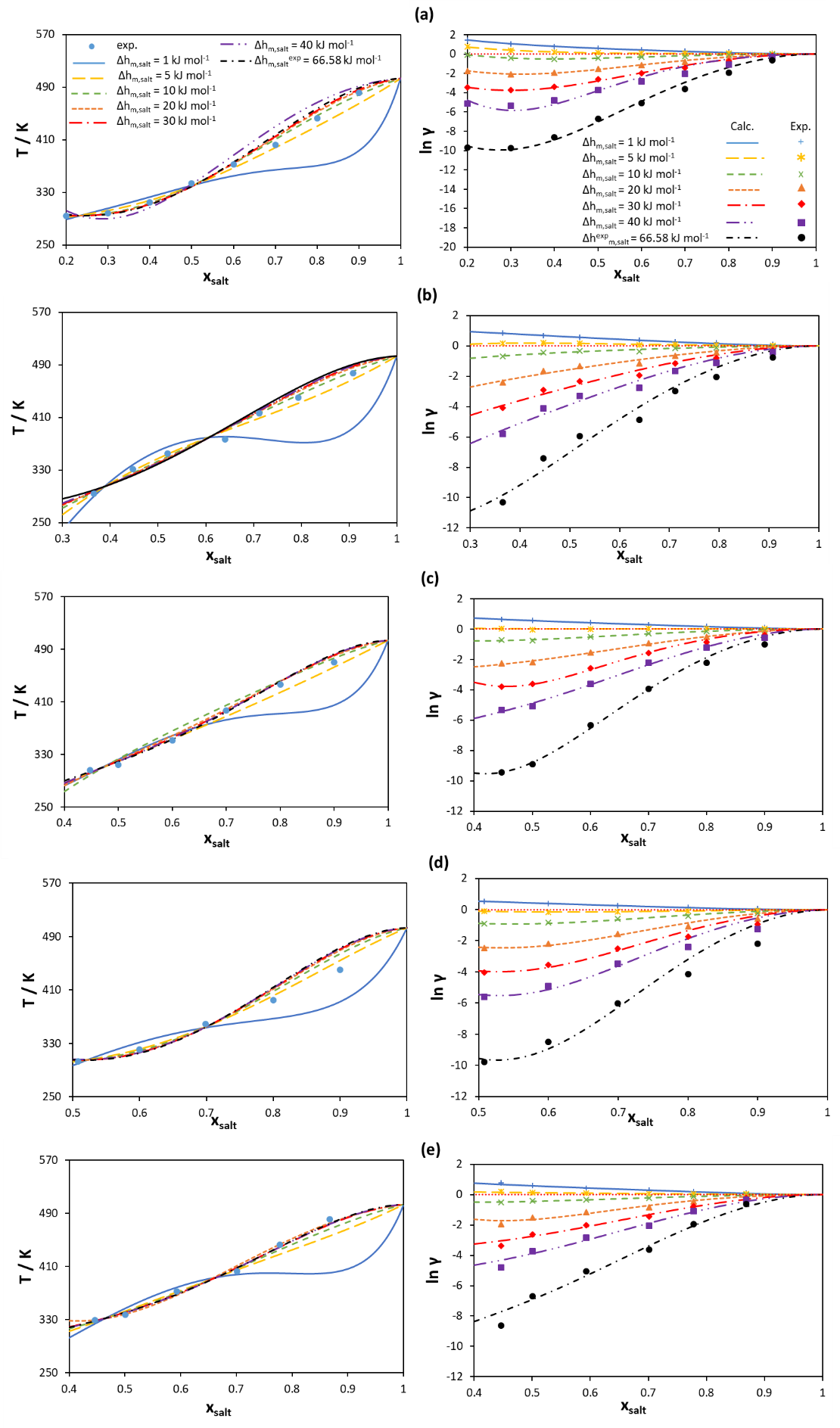


**Figure S7**. Liquidus line (left) and activity coefficients (right) of [N_3333_]Cl in binary mixture of [N_3333_]Cl and (a) capric acid (b) lauric acid (c) myristic acid (d) palmitic acid (e) stearic acid modeled using Redlich-Kister polynomial with three parameters assuming different melting enthalpy values. Experimental data are taken from [2].

**Table S2**. Empirical parameters of RK-polynomial for calculating activity coefficients of salts in binary mixtures of quaternary ammonium chloride salts and capric acid.

| Δh_m_ / kJ mol^−1^ | [N_1111_]Cl | | | [N_2222_]Cl | | | [N_3333_]Cl | | |
| --- | --- | --- | --- | --- | --- | --- | --- | --- | --- |
|  | a^(1)^ | b^(1)^ | c^(1)^ | a^(1)^ | b^(1)^ | c^(1)^ | a^(1)^ | b^(1)^ | c^(1)^ |
| 1 | 12.23 | -5.31 | -3.97 | 10.97 | -6.66 | -2.63 | 14.91 | -24.67 | 15.95 |
| 5 | 7.93 | -10.69 | 4.71 | 3.20 | -7.56 | 7.66 | 3.40 | -9.77 | 11.33 |
| 10 | -2.70 | -0.75 | 2.88 | -13.09 | 15.14 | -0.36 | -7.49 | -2.29 | 14.09 |
| 20 | - | - | - | -49.75 | 75.31 | -29.38 | -37.25 | 38.00 | 0.19 |
| 30 | -49.22 | 51.72 | -14.25 | -44.63 | -16.63 | 75.13 | -66.63 | 77.13 | -12.84 |
| 40 | -46.00 | -5.13 | 40.13 | -98.31 | 105.69 | -8.50 | -47.75 | -36.63 | 91.13 |
| Experimental | -32.75 | 44.56 | -19.59 | -96.88 | 18.38 | 95.00 | -170.38 | 208.75 | -51.88 |

**Table S3**. Empirical parameters of RK-polynomial for calculating activity coefficients of salts in binary mixtures of quaternary ammonium chloride salts and lauric acid.

| Δh_m_ / kJ mol^−1^ | [N_1111_]Cl | | | [N_2222_]Cl | | | [N_3333_]Cl | | |
| --- | --- | --- | --- | --- | --- | --- | --- | --- | --- |
|  | a^(1)^ | b^(1)^ | c^(1)^ | a^(1)^ | b^(1)^ | c^(1)^ | a^(1)^ | b^(1)^ | c^(1)^ |
| 1 | 26.33 | -48.76 | 20.49 | 10 | -18 | 19.5 | 12.13 | -5.17 | -9.59 |
| 5 | 8.63 | -10.90 | -9.88 | 0 | 0 | 4.5 | 2.68 | 1.91 | -7.20 |
| 10 | -10.63 | 23.44 | -33.81 | 0 | 0 | -13.5 | -10.48 | 16.09 | -9.34 |
| 20 | - | - | - | -35.5 | 62 | -48.25 | -42.06 | 65.38 | -33.56 |
| 30 | -124.94 | 325.88 | -305.63 | -68.5 | 116 | -76.75 | -72.50 | 110.19 | -53.63 |
| 40 | -108.25 | 153.25 | -98.25 | -110.5 | 205 | -138.75 | -104.75 | 161.63 | -79.38 |
| Experimental | -54.13 | 109.53 | -99.13 | -148.5 | 269 | -173.75 | -170.50 | 219.75 | -73.50 |

**Table S4**. Empirical parameters of RK-polynomial for calculating activity coefficients of salts in binary mixtures of quaternary ammonium chloride salts and myristic acid

| Δh_m_ / kJ mol^−1^ | [N_1111_]Cl | | | [N_2222_]Cl | | | [N_3333_]Cl | | |
| --- | --- | --- | --- | --- | --- | --- | --- | --- | --- |
|  | a^(1)^ | b^(1)^ | c^(1)^ | a^(1)^ | b^(1)^ | c^(1)^ | a^(1)^ | b^(1)^ | c^(1)^ |
| 1 | 12.83 | -3.81 | -9.66 | 17.84 | -28.73 | 14.94 | 19.55 | -39.47 | 24.72 |
| 5 | 8.74 | -15.07 | 7.05 | 6.02 | -14.03 | 12.09 | 1.45 | -6.17 | 6.78 |
| 10 | -0.93 | -12.67 | 13.61 | -6.00 | 10.00 | -10.00 | -17.99 | 20.95 | 0.38 |
| 20 | - | - | - | -41.50 | 72.00 | -43.00 | -66.80 | 120.27 | -61.87 |
| 30 | -31.69 | -32.06 | 65.25 | -79.00 | 139.00 | -78.50 | -68.44 | 5.09 | 111.50 |
| 40 | -77.50 | 67.94 | -4.25 | -108.00 | 176.00 | -88.00 | -161.44 | 305.31 | -171.47 |
| Experimental | -30.09 | 24.00 | 0.08 | -141.50 | 218.00 | -96.50 | -230.75 | 295.00 | -35.88 |

**Table S5**. Empirical parameters of RK-polynomial for calculating activity coefficients of salts in binary mixtures of quaternary ammonium chloride salts and palmitic acid.

| Δh_m_ / kJ mol^−1^ | [N_1111_]Cl | | | [N_2222_]Cl | | | [N_3333_]Cl | | |
| --- | --- | --- | --- | --- | --- | --- | --- | --- | --- |
|  | a^(1)^ | b^(1)^ | c^(1)^ | a^(1)^ | b^(1)^ | c^(1)^ | a^(1)^ | b^(1)^ | c^(1)^ |
| 1 | 20.54 | -29.42 | 7.66 | 17.55 | -33.27 | 22.19 | 14.81 | -24.81 | 11.25 |
| 5 | 6.47 | -9.34 | -0.71 | 0.29 | -5.85 | 12.72 | -11.00 | 23.20 | -6.38 |
| 10 | -11.72 | 18.42 | -13.98 | -21.98 | 31.63 | -2.62 | -45.94 | 97.00 | -45.47 |
| 20 | - | - | - | -46.50 | 23.03 | 51.75 | -116.00 | 245.31 | -124.25 |
| 30 | -89.75 | 151.44 | -89.13 | -109.28 | 172.75 | -53.94 | -185.75 | 392.38 | -202.00 |
| 40 | -126.50 | 208.69 | -117.63 | -158.56 | 267.22 | -104.25 | -257.00 | 545.88 | -286.00 |
| Experimental | -26.56 | -20.25 | 55.63 | -223.00 | 431.00 | -235.25 | -441.50 | 932.50 | -488.00 |

**Table S6**. Empirical parameters of RK-polynomial for calculating activity coefficients of salts in binary mixtures of quaternary ammonium chloride salts and stearic acid.

| Δh_m_ / kJ mol^−1^ | [N_1111_]Cl | | | [N_2222_]Cl | | | [N_3333_]Cl | | |
| --- | --- | --- | --- | --- | --- | --- | --- | --- | --- |
|  | a^(1)^ | b^(1)^ | c^(1)^ | a^(1)^ | b^(1)^ | c^(1)^ | a^(1)^ | b^(1)^ | c^(1)^ |
| 1 | 18.97 | -29.02 | 12.78 | 17.55 | -33.27 | 22.19 | 19.63 | -35.17 | 18.81 |
| 5 | 1.98 | -0.83 | -0.48 | 0.29 | -5.85 | 12.72 | 5.37 | -12.75 | 10.11 |
| 10 | -11.47 | 3.36 | 12.94 | -21.98 | 31.63 | -2.62 | -12.38 | 15.33 | -1.19 |
| 20 | - | - | - | -46.50 | 23.03 | 51.75 | -32.39 | 0.12 | 55.52 |
| 30 | -101.56 | 165.25 | -73.88 | -109.28 | 172.75 | -53.94 | -90.22 | 157.97 | -78.78 |
| 40 | -145.50 | 259.00 | -141.75 | -158.56 | 267.22 | -104.25 | -130.98 | 237.61 | -126.69 |
| Experimental | -61.25 | 98.13 | -41.81 | -196.75 | 301.19 | -87.13 | -239.94 | 452.41 | -257.81 |

T**able S7**. Melting properties of quaternary ammonium chloride and fatty acids. Data are taken from [2]

| Compound | T_m_ / K | Δh_m_ / kJ mol^−1^ | ΔS_m_ / J mol^−1^ K^−1^ |
| --- | --- | --- | --- |
| [N_1111_]Cl | 612.87 | 20.49 | 33.43 |
| [N_2222_]Cl | 526.78 | 51.24 | 97.27 |
| [N_3333_]Cl | 503.07 | 66.58 | 132.35 |
| Capric acid | 304.75 | 27.5 | 90.24 |
| Lauric acid | 317.48 | 37.83 | 119.16 |
| Myristic acid | 327.03 | 41.29 | 126.26 |
| Palmitic acid | 336.84 | 51.02 | 151.47 |
| Stearic acid | 343.67 | 61.36 | 178.54 |

References

[1] L. Fernandez, L.P. Silva, M.A.R. Martins, O. Ferreira, J. Ortega, S.P. Pinho, J.A.P. Coutinho, Indirect assessment of the fusion properties of choline chloride from solid-liquid equilibria data, Fluid Phase Equilib., 448 (2017) 9-14.

[2] P.V.A. Pontes, E.A. Crespo, M.A.R. Martins, L.P. Silva, C.M.S.S. Neves, G.J. Maximo, M.D. Hubinger, E.A.C. Batista, S.P. Pinho, J.A.P. Coutinho, G. Sadowski, C. Held, Measurement and PC-SAFT modeling of solid-liquid equilibrium of deep eutectic solvents of quaternary ammonium chlorides and carboxylic acids, Fluid Phase Equilib., 448 (2017) 69-80.
