# Supplementary figures and images for: Modeling of Solid–Liquid Equilibria in Deep Eutectic Solvents: A Parameter Study
Source: Molecules. 2019 Jun 25;24(12):2334. doi: 10.3390/molecules24122334 (PMC6631263; doi:10.3390/molecules24122334)

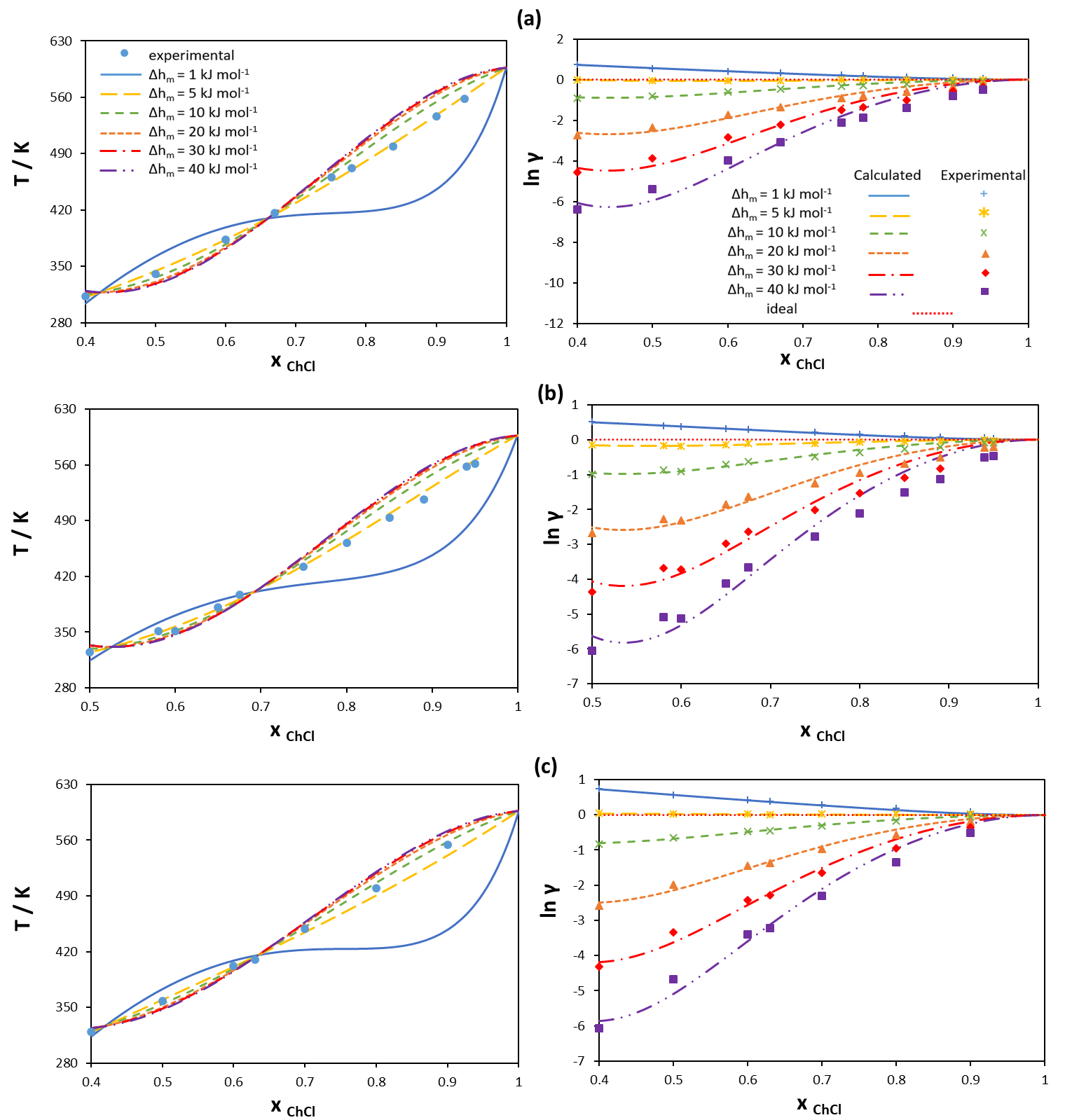

Supplement: Supplementary file 1 [file molecules-24-02334-s001.zip › supplementary/Figure S1.png]

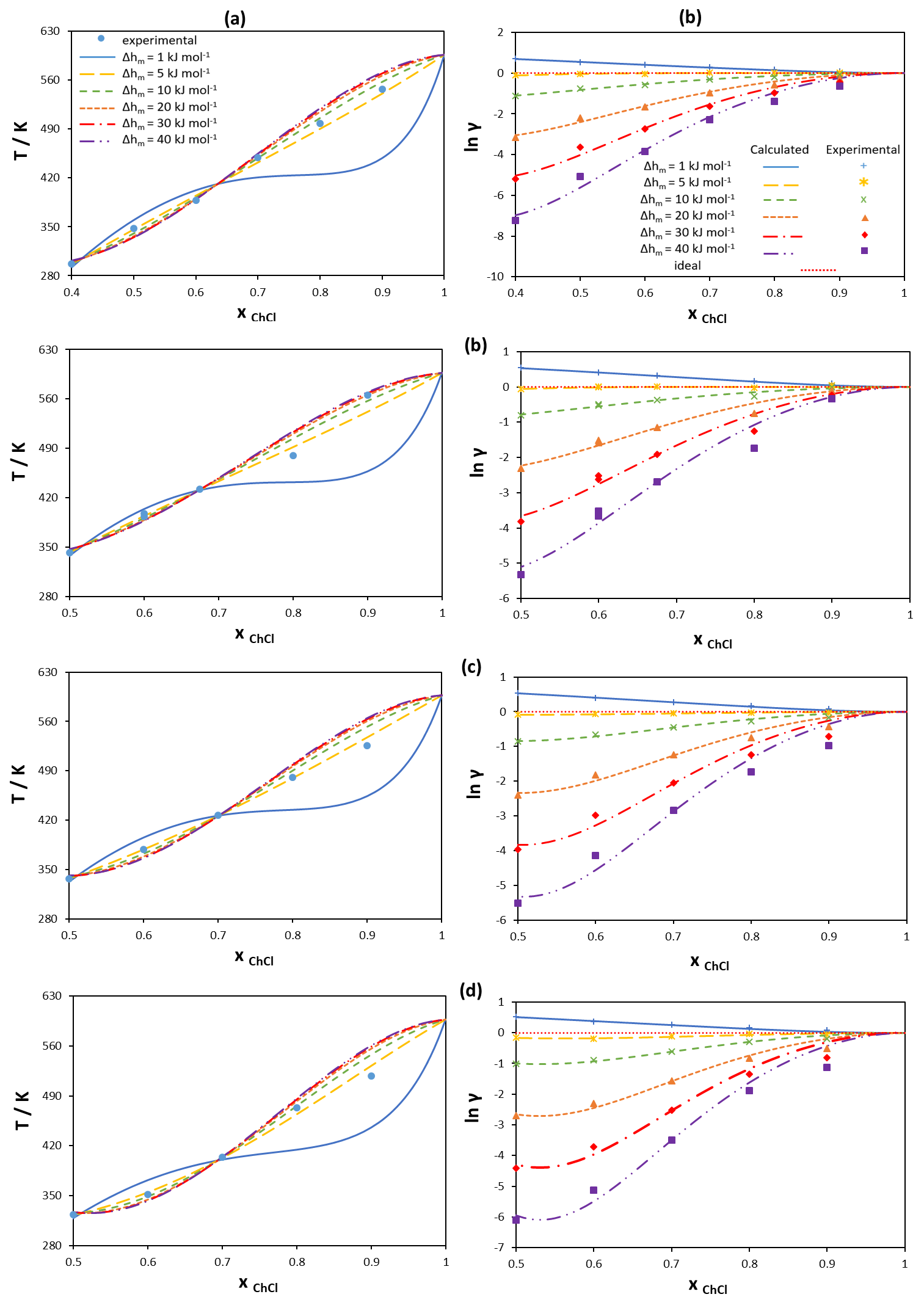

Supplement: Supplementary file 1 [file molecules-24-02334-s001.zip › supplementary/Figure S2.png]

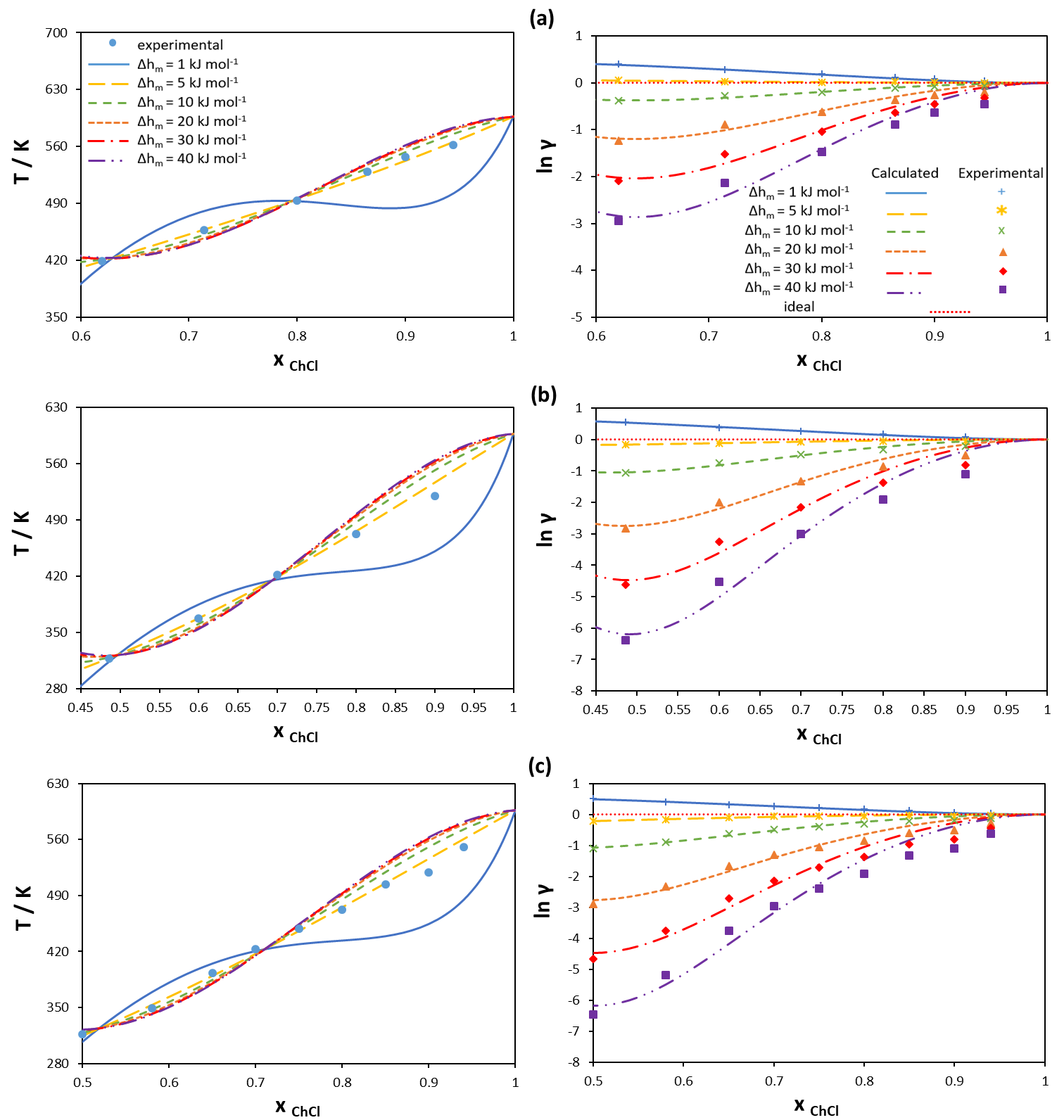

Supplement: Supplementary file 1 [file molecules-24-02334-s001.zip › supplementary/Figure S3.png]

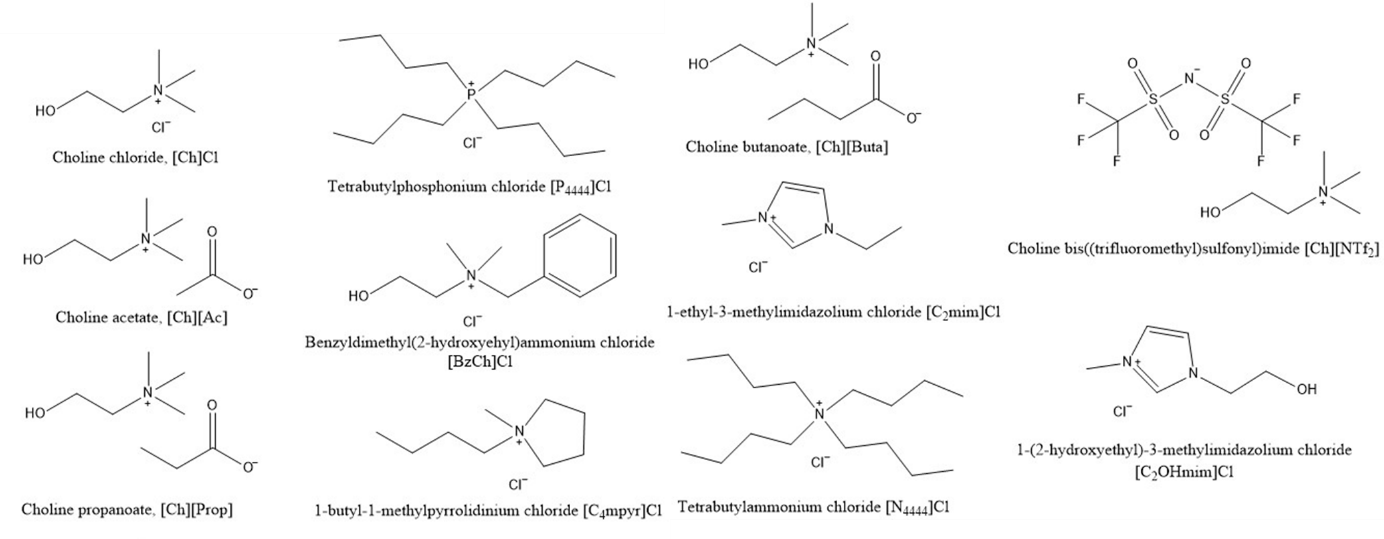

Supplement: Supplementary file 1 [file molecules-24-02334-s001.zip › supplementary/Figure S4.png]

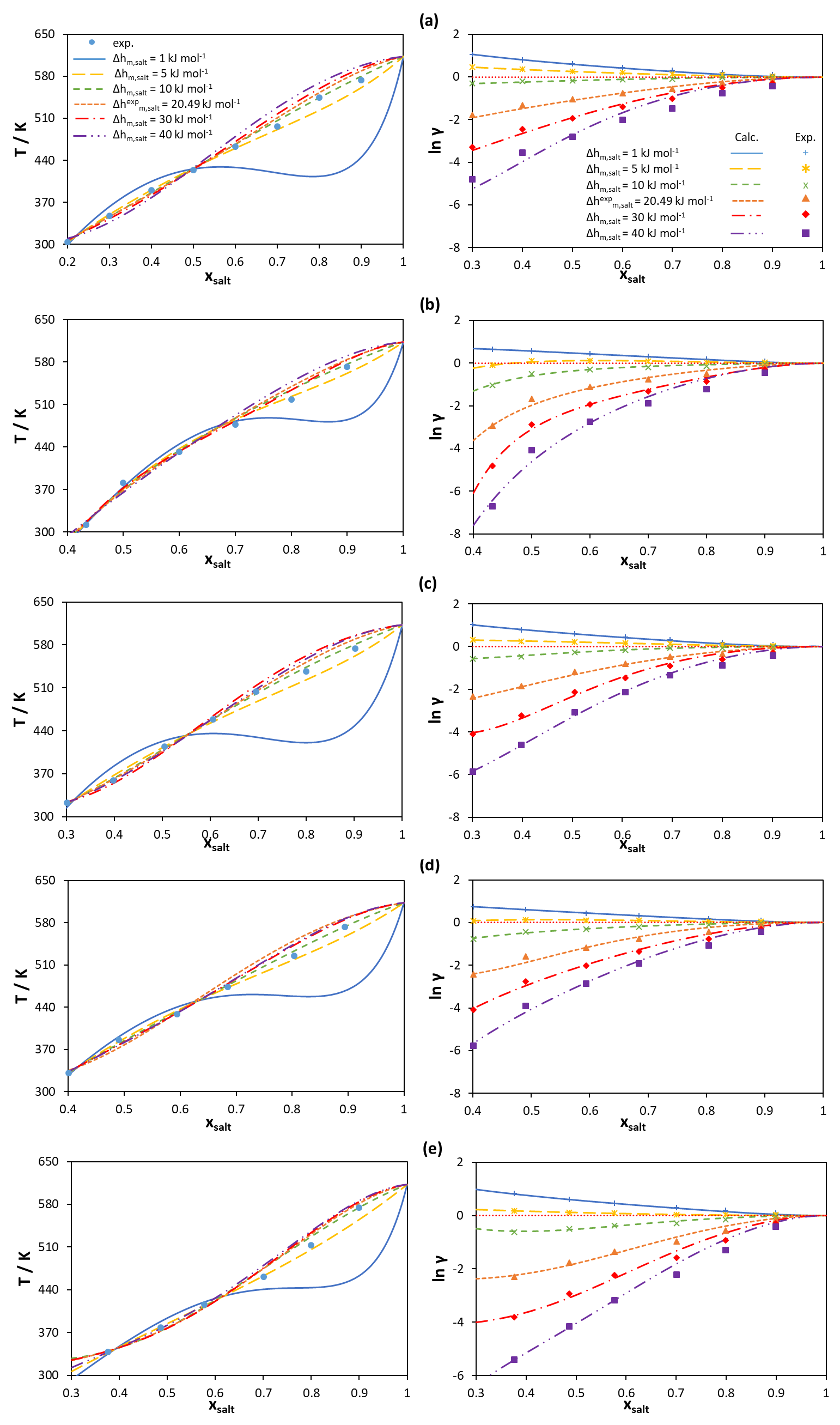

Supplement: Supplementary file 1 [file molecules-24-02334-s001.zip › supplementary/Figure S5.png]

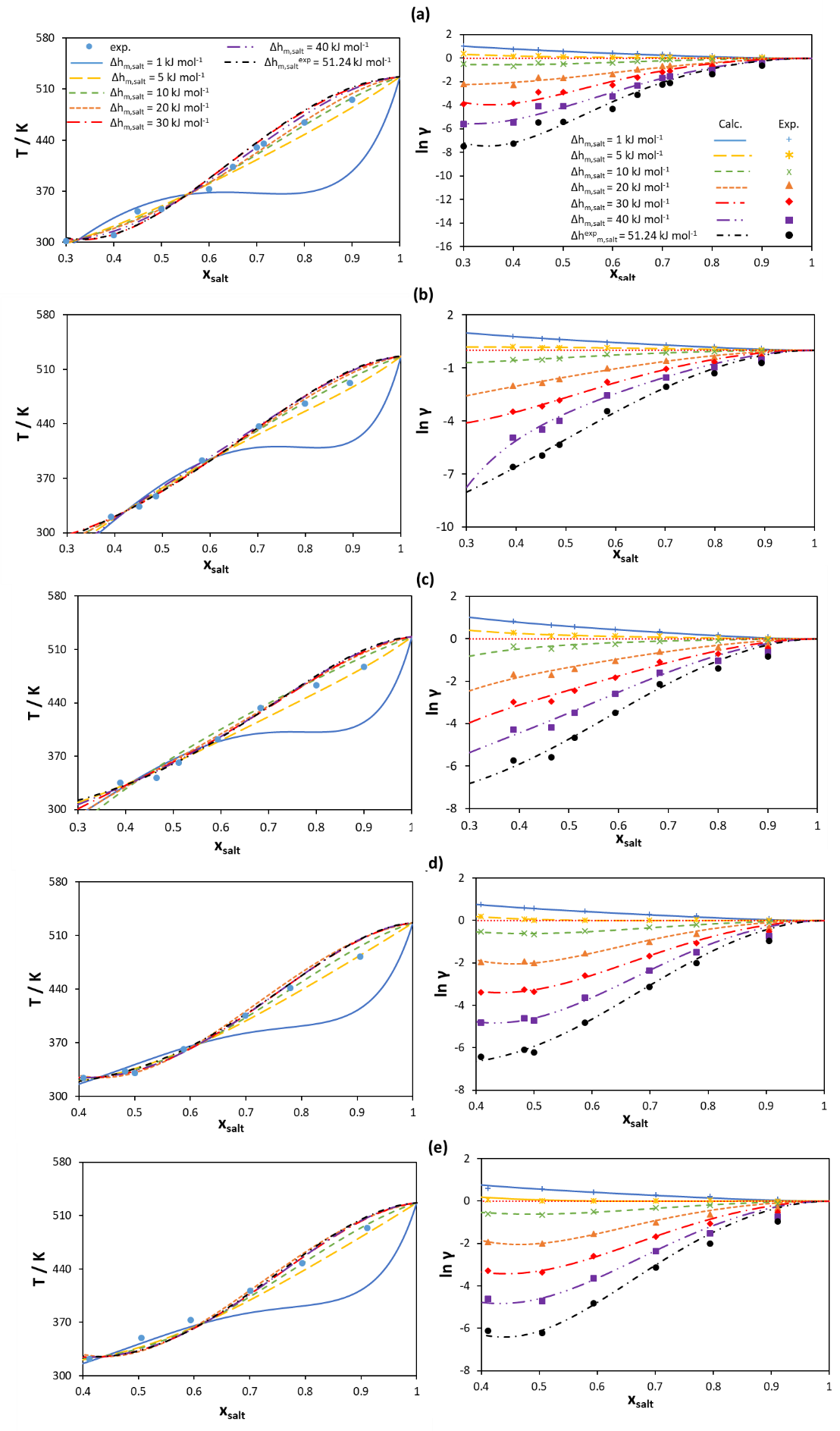

Supplement: Supplementary file 1 [file molecules-24-02334-s001.zip › supplementary/Figure S6.png]

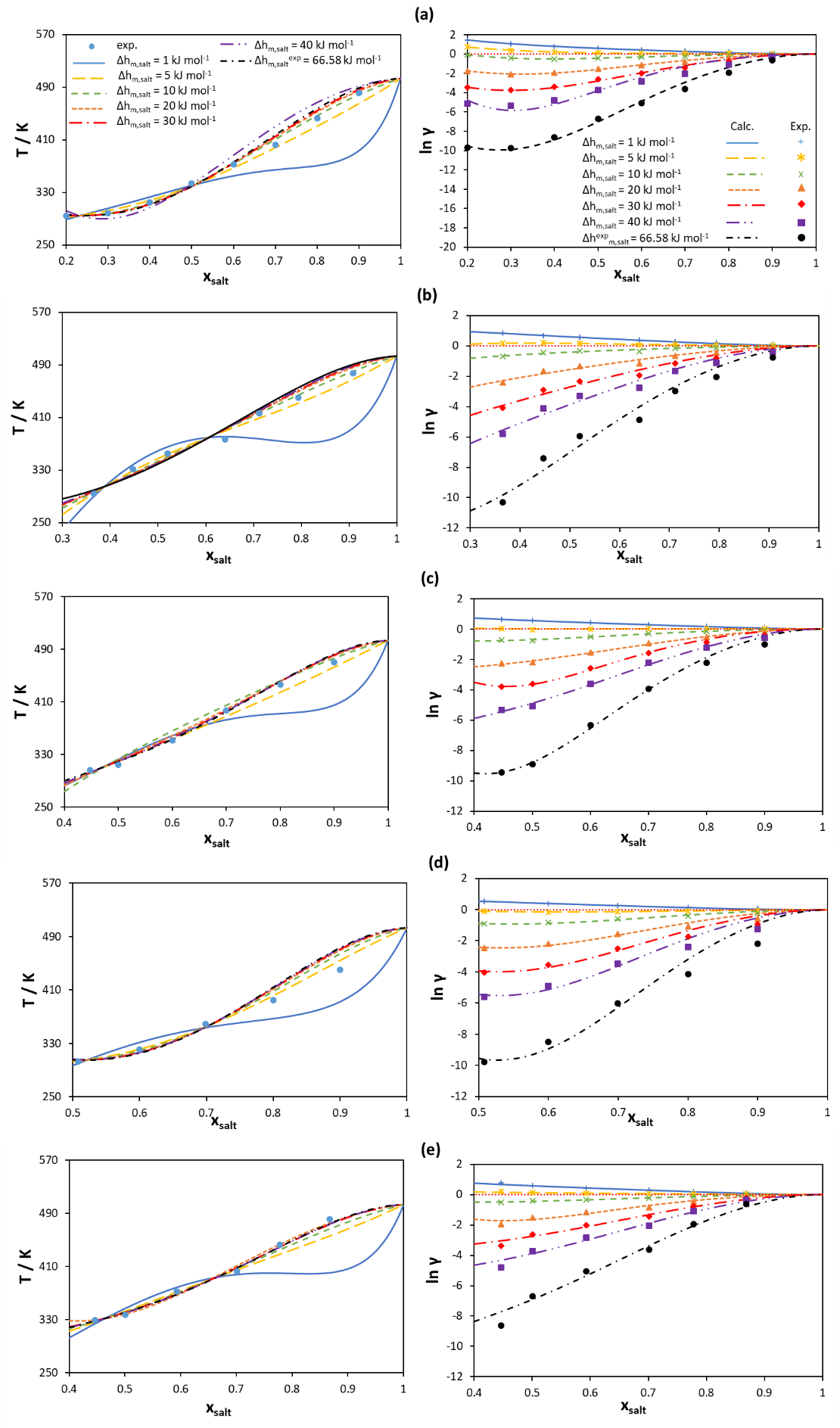

Supplement: Supplementary file 1 [file molecules-24-02334-s001.zip › supplementary/Figure S7.png]
